# Antioxidant potential of *Pediococcus pentosaceus* strains from the sow milk bacterial collection in weaned piglets

**DOI:** 10.1186/s40168-022-01278-z

**Published:** 2022-06-01

**Authors:** Leli Wang, Qihang Liu, Yuwei Chen, Xinlei Zheng, Chuni Wang, Yining Qi, Yachao Dong, Yue Xiao, Cang Chen, Taohong Chen, Qiuyun Huang, Zongzhao Zhai, Cimin Long, Huansheng Yang, Jianzhong Li, Lei Wang, Gaihua Zhang, Peng Liao, Yong-Xin Liu, Peng Huang, Jialu Huang, Qiye Wang, Huanhuan Chu, Jia Yin, Yulong Yin

**Affiliations:** 1 Hunan Provincial Key Laboratory of Animal Intestinal Function and Regulation, College of Life Sciences, Hunan Normal University, Changsha, China; 2 Institute of Subtropical Agriculture, Chinese Academy of Sciences, Changsha, China; 3 The National and Local Joint Engineering Laboratory of Animal Peptide Drug Development, College of Life Sciences, Hunan Normal University, Changsha, China; 4 State Key Laboratory of Plant Genomics, Institute of Genetics and Developmental Biology, Chinese Academy of Sciences, Beijing, China; 5 College of Animal Science and Technology, Hunan Agricultural University, Changsha, Hunan China; 6 Shandong Yihe Feed Co, Ltd, Yantai Hi-tech Industrial Development Zone, Yantai, Shandong China

**Keywords:** Culturomics, Sow milk, Probiotic, Oxidative stress, *Drosophila*, Piglets

## Abstract

**Background:**

In modern animal husbandry, breeders pay increasing attention to improving sow nutrition during pregnancy and lactation to favor the health of neonates. Sow milk is a main food source for piglets during their first three weeks of life, which is not only a rich repository of essential nutrients and a broad range of bioactive compounds, but also an indispensable source of commensal bacteria. Maternal milk microorganisms are important sources of commensal bacteria for the neonatal gut. Bacteria from maternal milk may confer a health benefit on the host.

**Methods:**

Sow milk bacteria were isolated using culturomics followed by identification using 16S rRNA gene sequencing. To screen isolates for potential probiotic activity, the functional evaluation was conducted to assess their antagonistic activity against pathogens *in vitro* and evaluate their resistance against oxidative stress in damaged *Drosophila* induced by paraquat. In a piglet feeding trial, a total of 54 newborn suckling piglets were chosen from nine sows and randomly assigned to three treatments with different concentrations of a candidate strain. Multiple approaches were carried out to verify its antioxidant function including western blotting, enzyme activity analysis, metabolomics and 16S rRNA gene amplicon sequencing.

**Results:**

The 1240 isolates were screened out from the sow milk microbiota and grouped into 271 bacterial taxa based on a nonredundant set of 16S rRNA gene sequencing. Among 80 *Pediococcus* isolates, a new *Pediococcus pentosaceus* strain (SMM914) showed the best performance in inhibition ability against swine pathogens and in a *Drosophila* model challenged by paraquat. Pretreatment of piglets with SMM914 induced the Nrf2-Keap1 antioxidant signaling pathway and greatly affected the pathways of amino acid metabolism and lipid metabolism in plasma. In the colon, the relative abundance of *Lactobacillus* was significantly increased in the high dose SMM914 group compared with the control group.

**Conclusion:**

*P. pentosaceus* SMM914 is a promising probiotic conferring antioxidant capacity by activating the Nrf2-Keap1 antioxidant signaling pathway in piglets. Our study provided useful resources for better understanding the relationships between the maternal microbiota and offspring.

Video Abstract

**Supplementary Information:**

The online version contains supplementary material available at 10.1186/s40168-022-01278-z.

## Introduction

The first year of life is a crucial stage of the development of the microbiome. Breastfeeding is a main factor in the development of the microbiome in this period [1]. Breastfed infants have a decreased risk of gastroenteritis and sudden infant death syndrome, because breast milk provides essential nutrients and a broad range of bioactive compounds for developing neonates [2]. Commensal bacteria from breast milk act as pioneer bacteria during the critical stage of initial neonatal gut colonization [3]. Several studies at the strain level have specifically demonstrated that there are some bacteria shared between human breast milk and infant feces by isolating and identifying bacteria from both sources [4]. The vertical transfer concept that the breast milk microbiota can be transmitted from mothers to infants during breastfeeding is now widely accepted [5]. The maternal microbiota contributes to the ‘initial’ intestinal microbiota establishment in infants and helps to modulate both short- and long-term infant health outcomes [6–8].

Due to the high degree of similarity in anatomy, physiology and immunology between humans and pigs, piglets have been extensively used as an ideal model to study neonatal gastrointestinal system and health [6]. Breast milk is the main food source for piglets prior to weaning. Early weaning is an abrupt event that often leads to severe oxidative stress in piglets and restricts the development of pigs immediately post-weaning in commercial swine husbandry. Although abundant probiotic bacterial strains have been isolated from human and bovine milk [9–11], strategies for the preservation and isolation of commensal bacteria from sow's milk are limited currently. While culture-independent methods have allowed an understanding of the composition and diversity of sow milk microbiota [12], culture-dependent methods are still critical for the functional identification and utilization of the sow milk microbiota. However, no research using culture-dependent methods has so far systematically investigated the sow milk microbiota which might be indirectly or directly beneficial for precise care of early-weaned piglets [13].

Lactic acid bacteria (LAB) have proven to be desirable and worth exploring in a wide range of fields [14], yet the main probiotic genera in food production and supplements are typically limited to *Lactobacillus* and *Bifidobacterium* [15]. To explore other potential probiotic species, we focused on *Pediococcus Pentosaceus*. *P. pentosaceus*, is a Gram-positive, catalase-negative and homofermentative bacterial species that has long been used as a biopreservative in commercial starters of fermented foods [16–18]. In recent years, it has shown probiotic potential including anti-inflammatory, antioxidant, and detoxification properties as well as antagonist activity against pathogens [19–23]. For instance, *P. pentosaceus* L1, selected from pickled radish, exhibited tolerance to gastrointestinal conditions and reduced expression of proinflammatory genes in porcine intestinal epithelial cells infected by *Escherichia coli* [24]. In addition, heat shock protein from cell walls of *P. pentosaceus* AK-23 functioned as a lipopolysaccharide neutralizing protein and decomposed lipopolysaccharide into fatty acids and sugars [22].

In this study, a collection of LAB from sow’s milk was first established by culturomics, and included 1240 isolates. We used antagonistic experiments against pathogens *in vitro* and antioxidant tests in *Drosophila* species to select the candidate probiotic. Interestingly, the antioxidant activity of *P. pentosaceus* SMM914 in piglets was also verified by the activation of the Nrf2-Keap1 pathway. The importance of sow milk bacteria deserves more attention for promoting healthy swine production.

## Results

### A collection of lactic acid bacteria from sow milk

**Fig. 1:**
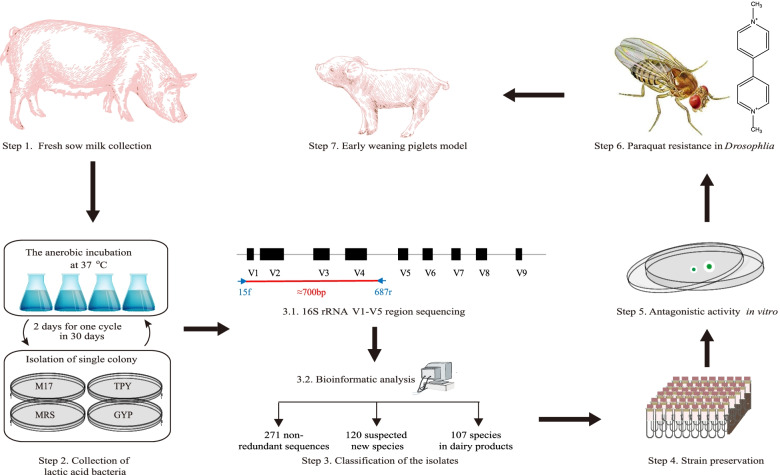
The workflow for large-scale bacterial cultivation from sow milk and the characterization of functions

Breast milk is a common source of probiotic strains [20]. We hypothesized that these LAB from sow’s milk could facilitate adaptive and functional changes that optimize the weaning transition in piglets, a time when they are vulnerable to various stressors. However, the diversity and function of bacteria in porcine milk remain relatively understudied. To establish a sow milk bacterial collection (smBC), a large-scale cultivation and identification of the sow milk microbiota (SMM) were performed by sow milk collection, anaerobic culturing and sequencing (Fig. 1, steps 1–3).

After the first three steps, we obtained 1240 isolates. These isolates were grouped into 271 bacterial taxa in the CD-HIT analysis based on clustering of sequences at 99% identity. The SILVA 16S rRNA database, NCBI nucleotide database and DAIRYdb reference gene database were used to classify these sequences into two categories: suspected new bacterial species and previously identified species. A phylogenetic tree was built based on the calculated distances between pairs of sequences (Suppl. Data S1). The results showed that 151 taxa were assigned to previously described species (black, Fig. S1). The alignment against the DAIRYdb revealed that 107 out of the 151 taxa could be assigned to species found in dairy products (red dots, Fig. S1). However, the other 120 taxa could not be assigned to any known species (blue, Fig. S1).

**Fig. 2:**
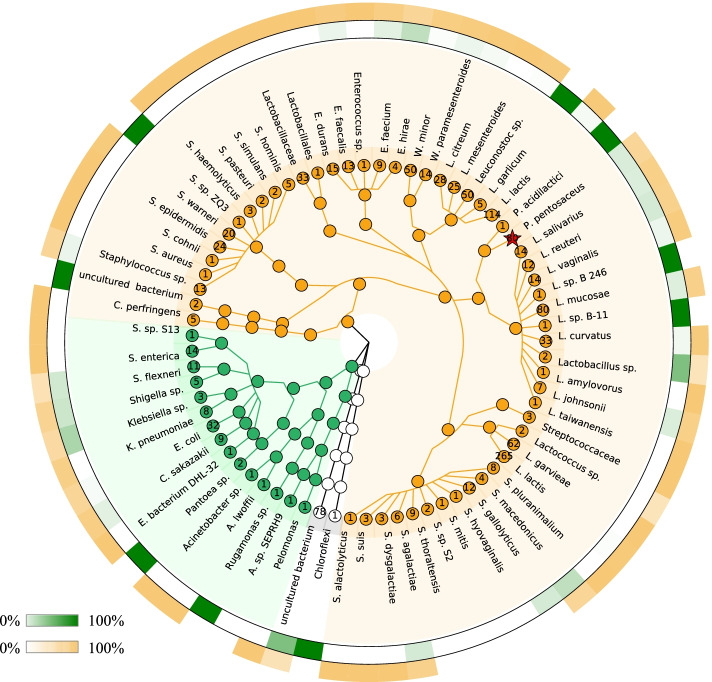
The sow milk bacterial collection. The inner circles depict taxonomic assignments for the bacteria that are isolated where the *Proteobacteria* were represented in green, and the *Firmcutes* in yellow. The taxa names are labeled, and the number of different taxa within each species is provided at the nodes. *P. pentosaceus* is indicated in red. The probability (%) of suspected new species and known species identified from the dairy products is shown in the outer rings with the green or yellow heat map, respectively

Specifically, twenty-three bacterial taxa were found and previously described in both the DAIRYdb and SILVA database or the NCBI nucleotide database, including *Acinetobacter lwoffii*, *Acinetobacter sp.*, *Clostridium perfringens*, *Pelomonas aquatic*, *Enterococcus sp.*, *Enterococcus durans*, *Lactobacillus amylovorus*, *Lactobacillus taiwanensis*, *Lactococcus garvieae*, *Leuconostoc mesenteroides*, *Salmonella sp*. and *Salmonella* Typhimurium. Nine isolates were from suspected new species and also could not be assigned to any known species in the DAIRYdb. In addition, five isolates were assigned to a previously described species in the SILVA database or NCBI nucleotide database, but not in the DAIRYdb, including *Acidovorax sp.* SEPRH9, *Streptococcus hyovaginalis*, *Streptococcus mitis*, *Streptococcus sp.* S2 and *Streptococcus thoraltensis* (Fig. 2). In total, 922 out of the 1240 isolates belonged to the *Lactobacillales* order (Suppl. Data S2), and this group was dominated by *Lactococcus lactis*. The genera staphylococci and streptococci represented 5.81% and 4.03% of the total bacterial isolates, respectively.

### Screening of candidate probiotic *P. pentosaceus* strains

We focused on *Pediococcus* spp. to explore a potential health-promoting bacterial genus that was not one of the traditional probiotic genera *Lactobacillus* or *Bifidobacterium*. Piglets commonly encounter pathogens on farms at increasing frequencies [25], including *Salmonella* Typhimurium [26], enterohemorrhagic *Escherichia coli* (EHEC) [27], enterotoxigenic *E. coli* (ETEC) [28], *Klebsiella pneumoniae* [29], *Aeromonas punctate* [30], *Staphylococcus aureus* [31], *Listeria monocytogenes* [2] and *C. perfringens* [32]. Antagonistic activity against pathogens to control their spread is an important selection criterion for potential probiotic strains for use in pigs [33]. In inhibition assays, the fluctuating size of the inhibition zone of various *P. pentosaceus* isolates against these pathogens revealed strain-specific antimicrobial activity against different bacteria. The morphology of the ten strains with the strongest antimicrobial activity was observed, and they were cocci-shaped in pairs or quadruplets (Fig. S2).

**Fig. 3:**
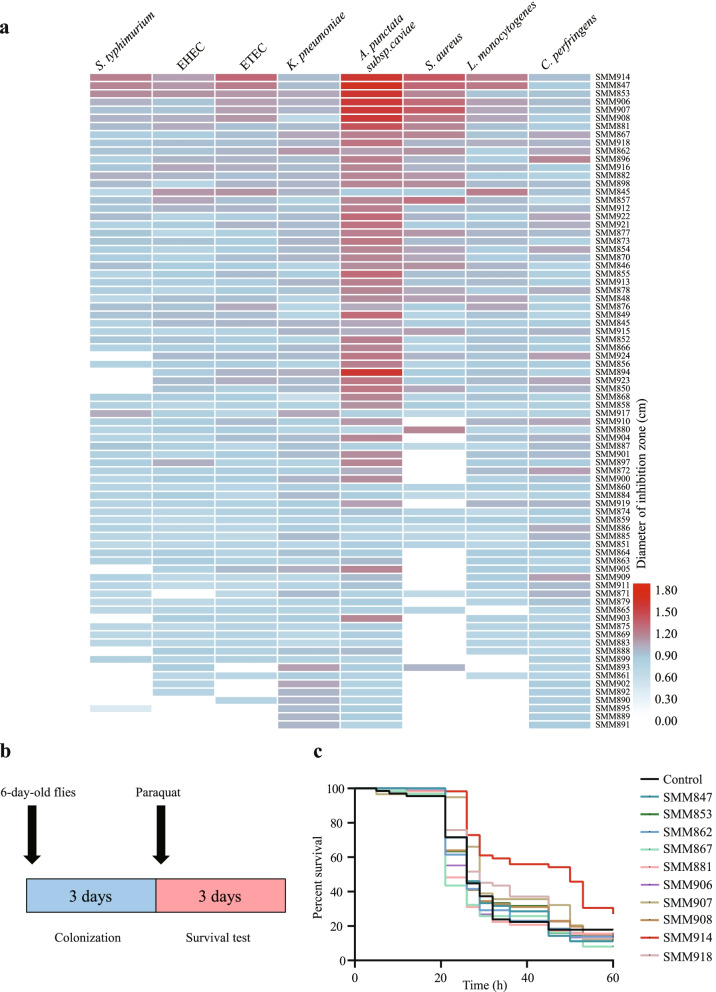
Screening of potential probiotic bacterial strains prior to a piglet feeding experiment. (a) Antagonistic activity against pathogens *in vitro*. (b) Graphical representation of experiments conducted to assess the survival rate in *Drosophila melanogaster w*^*1118*^. (c) *P. pentosaceus* confers protective effects upon *Drosophila* against paraquat. Statistical differences were calculated by the log-rank test

For these *P. pentosaceus* strains, we next used an animal model, *Drosophila melanogaster*, to rapidly screen bacteria that showed potent antioxidant activity *in vivo* (Fig. 3b). After paraquat treatment for 45 hours, the flies colonized with *P. pentosaceus* SMM914 showed a significantly elevated survival rate in response to paraquat challenge, compared to the control (*p* < 0.05, log-rank test) (Fig. 3c).

SMM914 showed a strong ability to inhibit the proliferation of *S. aureus*, while almost one-third of *P. pentosaceus* strains exhibited no antimicrobial ability toward this pathogen. After the sodium hydroxide neutralization reaction, the antibacterial effect of SMM914 against *S. aureus* and ETEC was abolished, which proved that its bacteriostatic effect was mainly due to the presence of organic acids (Fig. S3). The time curves for growth and pH of SMM914 were also measured. The results revealed that the strain entered the stationary phase after 12 hours of fermentation, while the pH was stabilized at approximately 3.9 after 36 hours of fermentation (Fig. S4). The genome of SMM914 was sequenced, which generated 28 contigs and a total size of 1,924,818 bp and a GC content of 37.14%. Through annotation of the SMM914, there were genes related to oxidative stress resistance, including organic acid, thioredoxin, and exopolysaccharide (Fig. S5, Suppl. Table S1 and Suppl. Data S3). Antibiotic resistance genes of SMM914 consist of bacitracin, tetracycline and erythromycin (Suppl. Table S2). A series of virulence determinants are predicted in SMM914 including capsule, lipopolysaccharide, ClpE, ClpC and ClpP (Suppl. Table S3), some of which are required for adhesion [34] and growth under stress conditions [35].

### Serum biochemical parameters in the pig-feeding trial

**Fig. 4:**
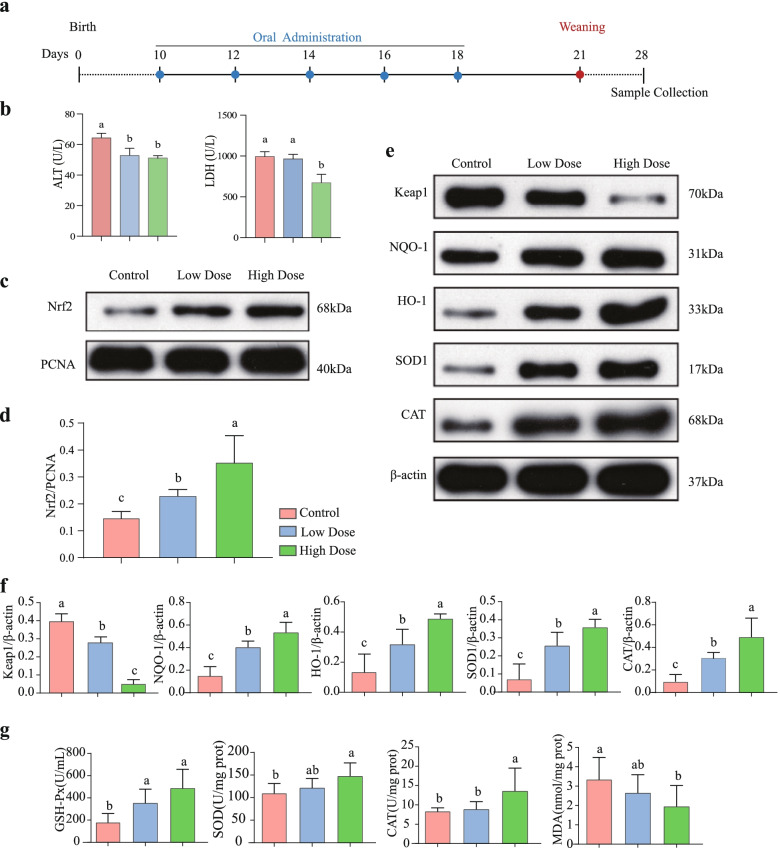
Effects of the oral administration of *P. pentosaceus* SMM914 on the antioxidation capacity and activation of the cytoprotective Nrf2 pathway in piglets. (a) Experimental outline in piglets (*n* = 18). Piglets were kept for 10 days after birth for adaptation. Oral administration of *P. pentosaceus* SMM914 was performed on days 10, 12, 14, 16 and 18 at low or high doses. At day 7 postweaning, seven piglets per treatment were randomly selected for slaughter for sample collection. (b) Alanine amiotransferase (ALT) and lactate dehydrogenase (LDH) were significantly decreased in piglets administered *P. pentosaceus* SMM914. (c-d) A graph and a bar chart of western blotting showing Nrf2 protein expression levels in the liver normalized to PCNA expression in the nucleus. (e-f) Western blotting analysis of antioxidant protein (Keap1, NQO-1, HO-1, SOD1 and CAT) levels in the liver and bar graph of data showing their protein expression normalized to β-actin expression in hepatic cells (*n* = 6). (g) The enzyme activities of GSH-Px, SOD, CAT and MDA were measured in liver lysates. Values of the bars stand for significant differences using one-way ANOVA followed by Duncan’s multiple comparisons at *p* <0.05. Data are the mean ± s.e.m, *n* = 7

Because *P. pentosaceus* SMM914 showed bacteriostatic activity and enhanced resistance to paraquat-induced stress in *Drosophila*, it was selected to feed the piglets in low-dose (LD) or high-dose (HD) groups prior to early weaning (Fig. 4a). Regarding visceral indices, a higher heart coefficient, which is the relative weight of the heart was observed in piglets treated with *P. pentosaceus* SMM914 (*p* < 0.05, *n* = 7) (Fig. S6). Because the heart coefficient is negatively associated with oxidative stress [36], we speculated that the increased heart coefficient observed in this study could be an indicator of alleviated stress. Results of serum biochemical parameters revealed that total protein (TP) and albumin (ALB) levels decreased in the LD group (Fig. S7). Interestingly, using *P. pentosaceus* SMM914, regardless of *P. pentosaceus* SMM914 concentration used, the concentration of the hepatic disease biomarker alanine aminotransferase (ALT) was reduced (Fig. 4b). Moreover, the serum concentration of lactate dehydrogenase (LDH), which is associated with liver, was also significantly decreased in the HD piglets. Thus, *P. pentosaceus* SMM914 administration in piglets led to alleviation of liver injury during weaning.

### Pretreatment of piglets with SMM914 induces the Nrf2-Keap1 antioxidant signaling pathway

To discover the molecular mechanisms that might underlie the antioxidant effect, we investigated the alteration of the nuclear factor (erythroid-derived)-like 2 (Nrf2) signaling pathway in the liver of piglets by western blotting analysis and enzyme activity assays. As expected, in the western blotting analysis of this study, the protein level of Kelch-like ECH-associated protein 1 (Keap1) was remarkably suppressed in piglets receiving *P. pentosaceus* SMM914 compared to the control group (*p* < 0.05). We found that *P. pentosaceus* SMM914 not only markedly increased the intranuclear protein expression level of Nrf2 (Fig. 4c-d, Fig. S8) but also led to elevated protein levels of NADPH quinineoxidoreductase-1 (NQO-1), catalase (CAT), hemeoxygenase-1 (HO-1) and superoxide dismutase (SOD) in a concentration-dependent manner (Fig. 4c-f).

In the enzyme activity assays, the HD group simultaneously had increased glutathione peroxidase (GSH-Px) activity, CAT activity and SOD activity (*p* < 0.05) in the liver (Fig. 4g). Additionally, the HD group showed a significant decrease in malondialdehyde (MDA) concentrations, an end product of lipid peroxidation, in the liver compared with the control group (Fig. 4g). The western blotting data combined with enzyme activity tests suggests that *P. pentosaceus* SMM914 is a promising probiotic strain that confers antioxidant capacity through activation of the Nrf2-Keap1 antioxidant signaling pathway in piglets.

### The altered pathways of amino acid metabolism and lipid metabolism in plasma

**Fig. 5.**
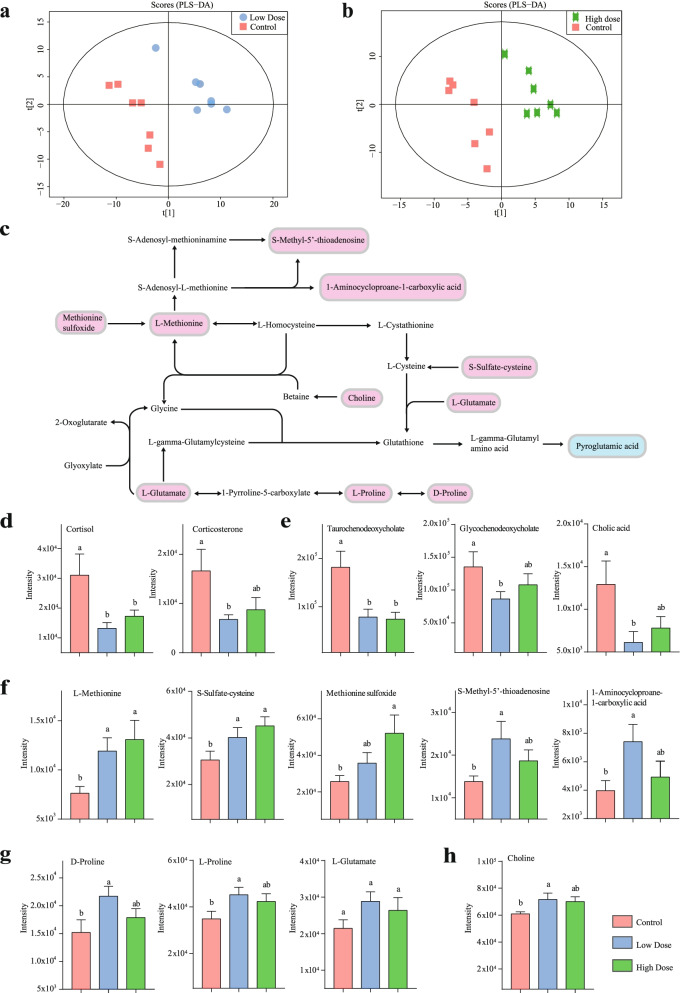
The metabolic profile of plasma. Score plots of the partial least squares discriminant analysis (PLS-DA) for the plasma metabolome (a) showing the scatter between the control and low-dose groups. (b) Scatter between the control and high-dose groups. (c) The integrative metabolism pathway according to the KEGG pathway database. Compared with the control group, the blue metabolites represent the intensities of metabolites that were downregulated, while the red metabolites represent the intensities of metabolites that were upregulated. (d-h) The perturbed metabolism pathways and metabolites in response to *P. pentosaceus* SMM914 treatment mainly include glutathione metabolism; cysteine and methionine metabolism; glycine, serine and threonine metabolism; arginine and proline metabolism; steroid hormone biosynthesis and primary bile acid biosynthesis. Data are the mean ± s.e.m, *n* = 7

To provide a better understanding of the antioxidant effect of *P. pentosaceus* SMM914, we further examined the metabolic profiles of blood plasma from the three groups (*n* = 7). PLS-DA plots showed separated clusters with an optimal goodness of fit (R^2^ = 0.996, Q^2^ = 0.681 (Fig. 5a); R^2^ = 0.994, Q^2^ = 0.479 (Fig. 5b)), indicating that the models were suitable and reliable for prediction. The KEGG enrichment of differential metabolites revealed that the pathways of lipid metabolism and amino acid metabolism were the main perturbed metabolic pathways between the HD group and the control group. The possible protective effect of *P. pentosaceus* SMM914 on the weaned piglets is depicted in Fig. 5c.

In the lipid metabolism, decreased levels of cortisol and corticosterone (Fig. 5d) were observed in plasma, which are classic stress hormones and related to steroid hormone biosynthesis. Meanwhile, the intensities of cholic acid, taurochenodeoxycholate and glycochenodeoxycholate (Fig. 5e) were also decreased in the LD group to 0.43-fold (*p* < 0.05), 0.58-fold (*p* = 0.08) and 0.47-fold (*p* < 0.05), respectively, compared to the levels in the control.

Conversely, in amino acid metabolism, several critical antioxidant metabolites (cysteine-S-sulfate, DL-methionine sulfoxide, L-methionine) closely related to cysteine and methionine metabolism were significantly increased to 1.41-2.03-fold in the HD group compared with the control group (*p* < 0.05) (**Fig.** **5****f**). D-proline, L-proline and L-glutamate, which are involved in arginine and proline metabolism, were increased in the LD group compared with the control group to 1.43-fold (*p* < 0.05), 1.30-fold (*p* < 0.05) and 1.34-fold (*p* = 0.056), respectively (Fig. 5g). In the glycine, serine and threonine pathway, choline was also significantly increased to 1.18-fold in the LD group (*p* < 0.05) compared with the control group (Fig. 5h). These three amino acid pathways are closely overlapped through several metabolites (Fig. 5c).

### The reshaped colon microbiota in piglets by SMM914

**Fig. 6:**
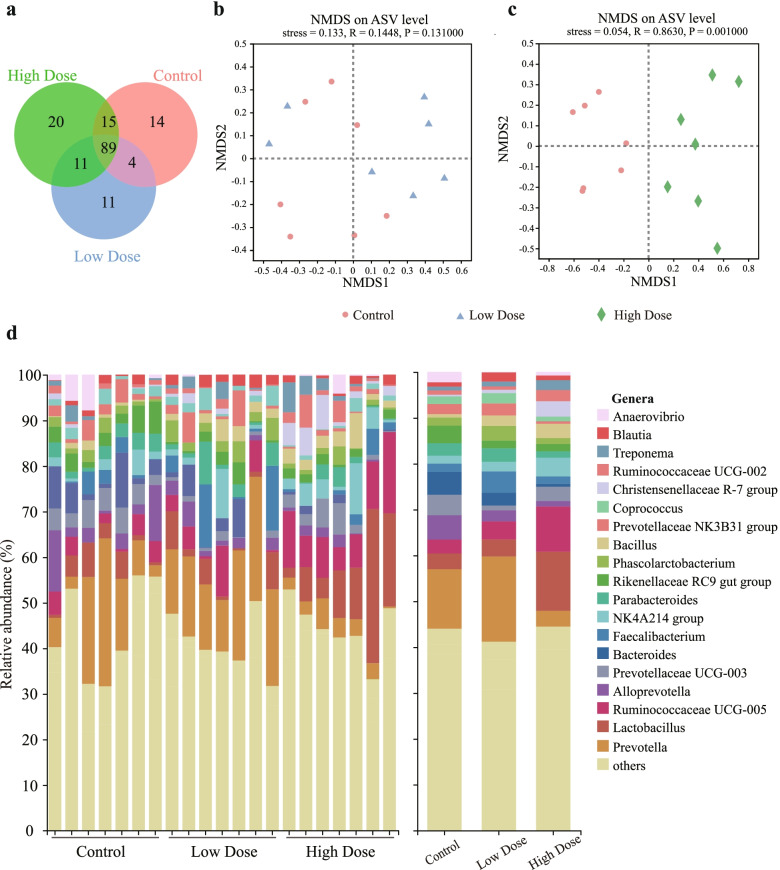
Microbial composition analysis in the colon. **a** The Venn diagram for ASVs among the control group, low-dose group and high-dose group. Scatterplots of NMDS analysis depicting differences in the bacterial community structure **b** between the control group and Low Dose group, and **c** between the control group and High Dose group. Analysis was performed using the weighted UniFrac phylogenetic distance metrics based on ASV level. Analysis of similarity (ANOSIM) was used for statistical testing of group similarities. **d** Individual (left) and averaged (right) taxon summary of bacterial genera in colon contents

Due to the changeover from milk to solid feed in weaned piglets, disorders in the composition of the gut microbiota can induce oxidative stress through a liver-gut axis [37–39]. In this study, the colonic microbiota was further investigated by using 16S rRNA gene amplicon sequencing. No differences were observed among the control and treated groups in terms of α-diversity (Fig. S9a). All samples from weaned piglets approached the saturation plateau based on Shannon-Wiener rarefaction curves (Fig. S9b), suggesting that the sampling was sufficient for nearly all bacterial species. The shared and specific genera are shown in a Venn diagram (Fig. 6a). The bacterial community of the three groups shared 89 genera. There were 20 genera unique to the HD group, including *Akkermansia*. Moreover, eleven genera, including *Dorea* and *Lachnospiraceae* AC2044 group, were detected in the LD and HD groups but not in the control group. To intuitively visualize the extent of the similarity of the overall bacterial community structure and composition, the results of NMDS (Fig. 6b-c) based on the weighted UniFrac distances revealed a separate clustering of samples from the HD group and the control group (stress = 0.054, R = 0.8630, *p* = 0.001, ANOSIM), but the samples from the LD group were not separated from the control group on ASV level (stress = 0.133, R = 0.1448, *p* = 0.131, ANOSIM).

The HD group showed distinct bacterial communities compared to the control group at both the family and genus level (Fig. S10 and Fig. 6d). For example, the relative abundance of the family *Lactobacillaceae* (*p* < 0.05) and the genus *Lactobacillus* (*p* < 0.05) were both increased in the HD group compared with the control group (Fig. S10b-c). For the families *Christensenellaceae* and *Ruminococcaceae*, which contain certain beneficial bacteria [40–44], the means of relative abundance in the LD group and HD group were increased by 89.57%, 110.27% and 804.9%, 21.59%, respectively, compared with the control group (Fig. S11). In addition, the genera *Christensenellaceae* R-7 group (*p* < 0.05) and *Ruminococcaceae* UCG-005 (*p* < 0.01) showed enrichment in the HD group (Fig. S11). Conversely, at the family level, the relative abundance of *Bacteroidaceae* in the HD group was decreased by 86.7% (Fig. S10). Specifically, the genus *Bacteroides* (*p* < 0.05) was observed to decrease in the HD group compared with the control group (Fig. S11).

## Discussion

In recent years, breeders have attached great importance to improving sow nutrition during pregnancy and lactation, rather than merely purchasing expensive creep feed for piglets at a later stage to effectively increase production and economic benefits. Here, we put forth the concept of Sow and Piglet Integration (SPI), which is an integral nutritional regulation scheme based on the physiological stages of sows and piglets on pig farms and the interrelationship of the microbiota between the two generations. Although the early weaning technique at 21 days is beneficial for sow productivity in intensive animal husbandry, this strategy leads to severe stress in piglets [45]. At this life stage, piglets experience a series of stressors, including separation from the mother, transport, the mixing of litters, diet transition and frequent exposure to potential pathogens, with a direct impact on breeders’ profitability [46]. To face challenges from pathogens and calls for reducing the use of antibiotics, some farmers in the United States have extended the lactation period to 25 days. Another promising alternative to antibiotics in animal feed is using probiotics to improve the absorption of nutrients [47]. During the initial development of mammalian neonates, breast milk is a nutritious food and a natural reservoir of probiotics [48, 49] that satisfies neonatal needs [50]. In the present work, we focused on the sow milk microbiota using culturomics. The domination of the order *Lactobacillales* in our collection was roughly consistent with the microbiota composition of sow milk during lactation according to a recent sequencing report [13]. The existence of bacterial isolates belonging to the genera staphylococci and streptococci also supported a view that commensal staphylococci and streptococci commonly occur in breast milk [51, 52] and may originate from the maternal skin [53]. This microbial identification work is an important step that revealed the LAB repertoire of sow milk, which contributes to further analyze the relationships between the maternal microbiota and that of the offspring.

In general, there are two main ways to mine bacterial strains and beneficial metabolites from a complex microbiome, which are (meta) genomics-based strategy and culture-based strategy [54]. Due to the increasingly lower sequencing costs, metagenomic strategy has already generated countless sequences, but some of sequences are hard to be assigned to living purified bacteria [55]. The culturomics strategy is an essential approach to verify the function of a purified bacterium and to illuminate the diversity of bacterial communities, which has been reported to double the species number of microorganisms isolated at least once from the human gut [56]. Yet, the culturomics strategy is time and labor consuming, and it is also a challenging task to evaluate the functional properties of such enormous isolates for researchers at strain level [57]. To screen potential probiotic strains from sow milk, we began with the evaluation of their antagonistic activity against pathogens *in vitro*. Next, we selected ten strains based on paraquat resistance assays to further explore their antioxidant capacities *in vivo*. Given the similarities of the intestinal development with mammals and the cost of the mouse model [58, 59], *Drosophila melanogaster* is an appropriate model to evaluate the ability of multiple bacterial strains to protect the host from reactive oxygen species (ROS), whose accumulation typically damages the health of both *Drosophila* and mammals [58, 60]. In this study, resistance to paraquat was used as a measure of free radical scavenging activity in the *Drosophila* system [60] for selecting potential probiotic bacterial strains.

Based on the results of antagonistic activity against pathogens and paraquat resistance assays, *P. pentosaceus* SMM914 was selected in the next swine trial. The piglet feeding trial indicated that consumption of *P. pentosaceus* SMM914 influenced organ parameters, as observed with the increase in heart coefficient. The heart coefficient has been reported to be negatively associated with oxidative stress via changes in the angiotensin II-aldosterone-brain natriuretic peptide [36]. Furthermore, weaning is frequently associated with liver injury and alters serum biochemical parameters related to liver function [61]. The liver is a target organ of stress in vertebrates and is involved in the secretion of bile salts, the phagocytosis of residual materials and the metabolism of proteins as well as detoxification [62]. Several species of *P. pentosaceus* have been previously proven to alleviate obesity, fatty liver, and detoxification [63, 64]. In the piglets treated with *P. pentosaceus* SMM914, the decreases in serum levels of TP, ALB, ALT and LDH were indicative of hepatic protection against oxidative stress [65]. The shift observed in our study was in accordance with another report stating that the administration of *P. pentosaceus* LI05 significantly prevented acute liver injury in rats with a decrease in TP and ALT concentrations [66].

Nrf2 is a conserved signaling pathway for regulating antioxidative activities across metazoans [67, 68]. Keap1 is a specific repressor of Nrf2 via tight binding. Antioxidant metabolites can contribute to the dissociation of the Keap1 and Nrf2 complex, promoting Nrf2 movement into the nucleus. Nrf2 is transferred from the cytosol to the nucleus, resulting in the coordinated transcriptional upregulation of a battery of antioxidant enzymes and detoxifying proteins [69]. In our study, the activation of the Nrf2-Keap1 signaling pathway in the liver of piglets was verified by western blotting analysis and enzyme activity assays. An existing body of literature describing the protective effects of probiotics against oxidative injury agrees with our findings. For example, *P. pentosaceus* ZJUAF-4 protected diquat-treated mice from oxidative stress-induced damage by modulating the Nrf2 pathway and gut microbiota [70]. Recently, a human commensal *Lactobacillus rhamnosus* GG strain was reported to stimulate Nrf2 in *Drosophila* liver analogs and the murine liver by 5-methoxyindoleacetic acid [71].

In plasma metabolism of this study, several metabolic pathways were perturbed, and they can be mainly classified into amino acid metabolism and lipid metabolism. In the cysteine and methionine pathway, L-methionine is a limiting amino acid in lactation stage associated with various key physiologic events [72]. The increased availability of L-methionine in early-weaned piglets was reported to have positive effects on plasma lipid metabolism and overall antioxidant status [73]. Methionine sulphoxide is biologically available as a methionine source through reductases [74]. High methionine bioavailability is likely to increase the entry of methionine into the one-carbon metabolism cycle, where S-Adenosyl-L-methionine, the ATP-activated form of methionine, is used to generate S-Methyl-5’-thioadenosine and 1-Aminocycloproane-1-carboxylic acid [75, 76]. Through the transsulfuration and transmethylation pathway, L-methionine could be converted into L-cysteine which serves as a key precursor for glutathione synthesis [77]. Glutathione is an endogenous sulfur-containing antioxidant and an effective scavenger of free radicals [78, 79]. Another substrate for glutathione synthesis is glutamate [80]. In mammals, glutamate is an abundant amino acid in milk that has proven to increase antioxidant enzyme activities [81, 82]. In the glutathione cycle, glutathione could be decomposed into L-gamma-glutamyl amino acid, and L-gamma-glutamyl amino acid is further converted to pyroglutamic acid [83]. A high level of pyroglutamic acid in serum reflects glutathione deficiency and is an indicator of the oxidative state [84]. While, in our study, the concentration of pyroglutamic acid was significantly downregulated. Collectively, the altered intensities of these metabolites might be conducive to the accumulation of glutathione. Moreover, the cysteine and methionine pathway is tightly overlapped with the glycine, serine and threonine pathway, because choline serves as the substrate for L-methionine synthesis (Fig. 5c). In the LD group of our study, the concentration of choline was markedly increased. Choline is widely regarded as an essential vitamin to regulate amino acid metabolism [85–87], particularly when L-methionine levels is not sufficient around parturition [88]. In pigs with intrauterine growth restriction, dietary supplementation with choline was reported to enhance the antioxidant capacity [89]. New evidence has also shown that choline deficiency-induced oxidative damage was associated with the generation of ROS and changes in Nrf2 signaling in the liver [90, 91]. Besides, in the arginine and proline pathway, excess glutamate can be used for proline biosynthesis [92, 93]. Proline is an essential amino acid for young pigs [94]. Our previous research found that oral administration of proline could improve mucosal proliferation and barrier function in piglets after stress injury [95].

In the lipid metabolism, decreased levels of cortisol and corticosterone were observed in this study (**Fig.** **5****d**), which are classic stress hormones and related to steroid hormone biosynthesis. It is worth noting that high stress could cause not only oxidative damage, but also the development of neurological disorders [82]. After maternal separation, separation anxiety in human infants is an inevitable phenomenon at weaning that may raise cortisol level and even alter the gut microbiota composition through the gut-brain axis [96, 97]. Hypersecretion of serum cortisol concentration is an indicator of excessive stress in pigs [98] and directly contributes to the pathology of anxiety [99]. Under psychological and emotional stress conditions, cortisol and corticosterone induce the oxidative load in the brain, with a significant increase in pro-oxidant markers in constantly changing environments [100, 101]. In future studies, we would pay attention to testing whether *P. pentosaceus* SMM914 can reduce the anxious-like behaviors in piglets, which were separated from sows, such as jumping against the walls and lying down. Additionally, in the primary bile acid biosynthesis pathway, the concentrations of cholic acid, taurochenodeoxycholate and glycochenodeoxycholate were decreased in groups treated by *P. pentosaceus* SMM914 (**Fig.** **5****e**). Variable derivatives of cholic acid and deoxycholic acid function as signaling molecules for the induction of oxidative stress [102, 103]. Sommerfeld *et al* reported that deoxycholic acid can combine with taurine or glycine to form taurochenodeoxycholate or glycochenodeoxycholate, which stimulates the phosphorylation of NADPH oxidase and the formation of ROS [104].

We observed that high-dose administration of *P. pentosaceus* SMM914 shaped the piglet colonic microbiota. The colonic microbiota of HD piglets was enriched with the *Lactobacillus*, *Christensenellaceae* R-7 group and *Ruminococcaceae* UCG-005 genera, while those of piglets in the control group exhibited higher relative abundances of genera from the *Bacteroidaceae* and *Prevotellaceae* families. The depletion of *Lactobacillus* spp. in the gut environment has been associated with oxidative damage, and various members of this genus have been commonly used as they may upregulate the expression of glutathione reductase and glutathione S-transferase during the suckling period [105, 106]. Moreover, methionine was reported to attenuate oxidative stress in rats through high abundances of *Lactobacillus* and *Lachnospiraceae* [107]. The *Ruminococcaceae* family is often negatively related to liver failure [41] and *Christensenellaceae* R-7 group plays a positive role in intestinal immunomodulation [108, 109]. On the contrary, members of *Bacteroidaceae* and *Prevotellaceae* have been proved to be associated with several diseases. For example, supplement of chitosan oligosaccharides in coronary heart disease patients could increase the antioxidant capacity by inhibiting the abundance of *Bacteroides* and *Prevotella* [106]. The dysbiotic microbiota in inflammatory bowel disease (IBD) patients is mostly characterized by an increase in *Prevotellaceae* and a reduction in *Ruminococcaceae* [110]. Improving the cellular antioxidant potential is a promising approach for prevention of IBD. In this work, it appeared that *P. pentosaceus* SMM914 administration in piglets positively regulated intestinal microbiota during the transition of early-weaned stress.

## Conclusion

Here, we focused on isolating LAB from sow milk using culturomics and established a set of methods for rapidly screening for potentially beneficial bacterial strains. In terms of the probiotic properties of the novel *P. pentosaceus* derived from sow milk, during the swine feeding trial, *P. pentosaceus* SMM914 administration appeared to alleviate potential liver injury during weaning by reducing the serum levels of ALT and TP. Meanwhile, it was also indicated that *P. pentosaceus* SMM914 could increase the heart coefficient of piglets, activate the Nrf2-Keap1 pathway in the liver and stimulate the levels of amino acid metabolites in plasma and the beneficial microbiota in colon. Overall, though we cannot provide comprehensive mechanistic linkages, this study has expanded upon the understanding of *P. pentosaceus* probiotic potential in piglets, and shed light on the importance of the sow milk microbiota for better understanding the relationships between the sow and offspring in biomedical research and agriculture.

## Methods

### Culture media and bacterial strain isolation

A total of nine healthy second-parity sows with similar breeding dates raised on a pig breeding farm (Changsha, China) were employed in this study. The sows received no antibiotics within the 4 weeks prior to breast milk sampling. The areolar skin around the teats was successively swabbed with alcohol (75%) tampons and warm saline-lubricated sterile swabs. Using sterile tubes, fresh milk was collected from six sows during lactation (**Fig.** **1****, step 1**).

Considering that bacterial populations can survive through cell death and recycling of dead cells [111], continuous culture and intermittent sampling were performed for 30 days in an anaerobic incubator (N_2_ = 90%, CO_2_ = 5%, and H_2_ = 5%) at 37°C (**Fig.** **1****, step 2**). Using sow milk as an inoculum, de Man, Rogosa, and Sharpe (MRS) (Oxoid, Code# CM0359, UK) [112], M17 (Oxoid, Code# CM0817, UK) [112], trypticase phytone yeast extract (TPY) (Hopebio, Code# HB0397, China) [113] and glucose yeast extract peptone (GYP) (Hopebio, Code# HB8539, China) [114] broth were utilized to cover as much LAB diversity as possible. The bacterial cells were harvested every other day by centrifugation at 4000 g for 10 min, and the cell pellets were resuspended in sterile normal saline. Then, the cells were spread on agar plates and anaerobically cultured in a DG250 Anaerobic workstation (DWS, UK) at 37°C for 24-72 hours. These isolates were first selected based on morphology including their size, separation from other colonies, ovality, color, halo-forming or not and the fuzziness or sharpness of the outline on plates. Colonies were re-streaked on agar plates, and all isolates were stocked in 25% (v/v) glycerol broth at -80°C at the College of Life Science, Hunan Normal University, China (**Fig.** **1****, step 4**).

### Characterization and classification of the isolated bacteria

After isolation and purification, DNA was extracted from pure cultures. The V1-V5 region of the 16S rRNA gene was amplified using Takara PrimerSTAR Max DNA Polymerase with a pair of LAB-specific primers, 15f (5′- GCTCAGGAYGAACGCYGG -3′) and 687r (5′- CACCGCTACACATGRADTTC-3′) for the identification of the isolates [115]. The PCR-amplified products were sequenced by Sanger sequencing (Sangon Biotech Ltd., China). The sequencing error-prone areas (50 bp) at both ends were removed. The partial 16S rRNA gene sequences were aligned against the NCBI nucleotide collection (nr/nt) database using BLASTN. The best match for each sequence was selected based on the smallest e-value (**Fig.** **1****, step 3**). The nonredundant set of 16S rRNA gene sequences was clustered by using CD-HIT version 4.7 with a sequence identity of 0.99 [116]. The phylogenetic relationship between isolates was determined by aligning the nonredundant set of 16S rRNA gene sequences to construct a maximum-likelihood tree by using FastTree version 2.1.7.

All 16S rRNA sequences were aligned against the SILVA database, the NCBI nucleotide database and the DAIRYdb reference database using BLASTN with a threshold of 1e-5 e-value, 99% coverage and 99% identity. The one with the smallest e-value in blast results was selected as best match. A 16S rRNA gene sequence was suspected to be from a new species when it had less than 99% identity with sequences in these databases including Silva version 132 16S rRNA database and NCBI nucleotide collection (nr/nt) database [116]. The sequencing read data have been deposited in the National Center for Biotechnology Information Sequence Read Archive (**Suppl. Data** **S1**).

The *P. pentosaceus* SMM914 genome was sequenced at the Beijing Tsingke Biotechnology Co., Ltd., using a NEBNext® Ultra™ DNA Library Prep Kit for Illumina (NEB, USA) following manufacturer’s recommendations. Briefly, more than 1 μg genomic DNA was extracted for sequencing library construction. The DNA sample was fragmented by sonication with a size of 350bp, and then, DNA fragments were end-polished and ligated with the full-length adaptor for Illumina sequencing with further PCR amplification. Next, the Illumina pair-end library was sequenced using an Illumina NovaSeq 6000 instrument with PE150. After quality control by our laboratory own compiling pipeline, paired reads were assembled using the SOAP denovo (version 2.04) [117, 118], SPAdes (version 3.15.1) [119] and ABySS (version 2.1.5) [120] into scaffolds. The CISA software (version 1.3) [121] was applied to integrate the assembly results of the three softwares. The assembly result with the least number of scaffolds was selected. The ab initio prediction method was used to get gene models and predict gene functions [122]. Gene models were identified using GeneMarkS (version 4.30) [123]. A whole genome Blast [124] search (E-value less than 1e-5, minimal alignment length percentage larger than 40%) against databases, including the Pfam Protein Database [125], Virulence Factors of Pathogenic Bacteria Database [126] and Antibiotic Resistance Genes Database [127]. *P. pentosaceus* SMM914 was deposited at the China General Microbiological Culture Collection Center (CGMCC20160).

### Antibacterial assay

The following indicator pathogenic strains were used: *S.* Typhimurium ATCC 14028, EHEC ATCC 43894, ETEC O149: K88, *K. pneumoniae* ATCC 13883, *A. punctata subsp. Caviae* ATCC 15468, *S. aureus* ATCC 25923, *L. monocytogenes* ATCC 19115 and *C. perfringens* ATCC 13124.

Agar well diffusion assays [128] were utilized to evaluate antimicrobial activity against the indicator pathogenic strains *in vitro*. Briefly, pathogens were grown in Luria-Bertani (LB) broth at 37°C for 8 hours and then diluted at a volume ratio of 20 μL to 4 mL LB and mixed well. Fifty microliters of the diluted liquid were spread evenly on each soft LB agar plate containing 0.8% agar. The residual liquid was evaporated on a ventilated clean bench. Next, holes were punched in each agar plate via sterile iron pipettes, with a depth of 6 mm and a diameter of 5 mm. The *P. pentosaceus* colonies were cultured on MRS broth at 37°C for 18 hours. Then, culture supernatants of *P. pentosaceus* strains were precisely added to the holes with a 30 μL volume per well. The central well of each plate was filled with 30 μL MRS broth as the negative control. After 48 hours of incubation at 37°C, antibacterial activity was observed as a halo of inhibition in the bacterial lawn formed around the sample, and the diameter of the zones of inhibition was measured. The evaluation of each sample was repeated in triplicate.

### Paraquat resistance assays in *Drosophila*

The *Drosophila* species was raised under a 12 h light:12 h dark cycle at 25°C on cornmeal-molasses medium. Six-day-old mated female *Drosophila melanogaster w*^*1118*^ were collected under CO_2_ anesthesia and starved for 2 hours. Each group consisted of 3 vials, and each vial contained 20 female flies. In the bacterial association assays, the colony-forming units (CFUs) of *P. pentosaceus* colonies were enumerated on MRS agar plates following standard microbiological procedures. Groups of adult female flies were colonized with pure cultures (1×10^10^ CFUs) of the *P. pentosaceus* strains for 3 days, including SMM847, SMM853, SMM862, SMM867, SMM881, SMM906, SMM907, SMM908, SMM914 and SMM918 isolates. Distilled water without *P. pentosaceus* was used as a negative control. Then, these flies were transferred to vials containing 2 pieces of Whatman paper soaked with 200 μL 5% (w/v) sucrose containing 12 mmol/mL paraquat (methyl viologen dichloride, Cat# 856177, Sigma-Aldrich, USA). Each group was supplied with fresh paraquat vials every day. The 12 mmol/mL concentration was chosen because it was empirically shown that this concentration was lethal to more than 80% of female *w*^*1118*^ flies within 2 days.

### Piglet feeding trial and sampling collection

The Animal Care and Use Committee of the Institute of Subtropical Agriculture, Chinese Academy of Sciences, reviewed and approved the experimental procedures involving piglets. As described in Jun Hu *et al* [115], a total of 54 newborn suckling piglets (Landrace × Yorkshire, *n* = 18) were chosen from nine second-parity sows and randomly assigned to three treatments among each litter, including a control group (physiological saline, 2.0 mL each time, Control), a low-dose SMM914 solution group (10^8^ CFU/mL, 2.0 mL each time, LD group) and a high-dose SMM914 solution group (10^9^ CFU/mL, 2.0 mL each time, HD group). The solution of saline or bacterial cells was infused into each piglet's mouth by a syringe without a needle. All suckling piglets were subjected to oral gavage every other day from the age of 10 to 18 days and were weaned at 21 days.

Seven litters were randomly selected from 9 litters at 28 days, and piglets from each treatment group and each litter were euthanized for sampling. Ten milliliters of blood were collected into heparin sodium anticoagulant tubes via direct cardiac puncture immediately after death and subjected to untargeted metabolism analyses. Another 10 mL of blood was collected in vacuum tubes and centrifuged at 3000 rpm at 4°C for 10 min. The serum samples were kept at -80°C until analysis. After the opening of the abdomen, tissues including the liver, spleen, kidney, and heart were weighed, dissected, and snap-frozen in liquid nitrogen. Colon contents were stored at -80°C until the extraction of bacterial DNA.

Serum concentrations of parameters reflecting lipid (cholesterol, triglyceride, high-density lipoprotein cholesterol, low-density lipoprotein cholesterol, lipase), protein (total protein, TP; albumin, ALB), carbohydrate (glucose, alpha-amylase, lactic dehydrogenase) metabolism, liver functionality including total bilirubin (TBIL), direct bilirubin (DBIL), indirect bilirubin (IBIL), the activity of aspartate transaminase (AST), alanine aminotransferase (ALT), gamma-glutamyl transpeptidase (GGT), serglobulin (GLO), alkaline phosphatase (ALP), lactic dehydrogenase (LDH) and cholinesterase (CHE), as well as kidney functionality including creatinine (CREA), blood urea nitrogen (BUN) and uric acid (UA) were determined using commercial kits according to the manufacturer’s instructions (Jiancheng Bioengineering Institute, Nanjing, China) and were identified with a TBA-120FR Automatic Chemistry Biochemistry Hiiometer (Hitachi Co., Tokyo, Japan).

### Western blotting analysis

Western blotting was performed as previously described with some modification [105]. Liver samples (*n* = 6) were powdered under liquid nitrogen and lysed in radioimmunoprecipitation assay buffer with the protease inhibitor phenylmethanesulfonyl fluoride (Beyotime Biotechnology). The supernatant was obtained by centrifugation at 12,000 × g for 10 min at 4°C. The denatured proteins were separated by 10% sodium dodecyl sulfate-polyacrylamide gel electrophoresis and then transferred to polyvinylidene fluoride membranes at 200 mA for 1 hour. The membranes were blocked with 5% nonfat milk in Tris-buffered saline mixed with 0.5% Tween-20 (TBST) at room temperature for 2 hours and then incubated with antibodies against Kelch-like ECH-associated protein 1 (Keap1) (SC-19917, Proteintech, USA), nuclear factor (erythroid-derived)-like 2 (Nrf2) (SC-98974, Proteintech, USA), NADPH quinineoxidoreductase-1 (NQO-1) (11451-1-AP, Proteintech, USA), hemeoxygenase-1 (HO-1) (27282-1-AP, Proteintech, USA), catalase (CAT) (66765-1-Ig, Proteintech, USA), Cu/Zn-superoxide dismutase (SOD1) (10269-1-AP, Proteintech, USA), PCNA (60097-1-Ig, Proteintech, USA) or β-actin (SC-47778, Proteintech, USA), which were diluted with 5% nonfat milk in TBST. The membranes were washed 3 times in TBST and then incubated with a secondary antibody. Finally, the membranes were washed with TBST and visualized with a chemiluminescence instrument.

Besides, on the cellular, the human hepatoma cells (HepG2) and the intestinal porcine enterocyte cell line (IPEC-J2) cells were selected to determine the antioxidant effects of fermentation liquid of *P. pentosaceus* SMM914. Cells were cultured in complete Dulbecco’s modified Eagle medium (DMEM) with 10% fetal bovine serum. MRS was used as the negative control (*n* = 3). *P. pentosaceus* SMM914 was grown in MRS broth at 37°C without shaking for 24 hours prior to centrifuged at 3000 × g for 5 minutes. Supernatants were collected from *P. pentosaceus* SMM914 fermentation broth and added in the DMEM at the volume ratio of 1%. After being washed twice in phosphate buffer saline, cells were exposed to the *P. pentosaceus* SMM914 treatment or MRS for 3 hours. Nuclear extracts of HepG2 and IPEC-J2 were fractionated by a Nuclear Protein Extraction Kit (Beyotime Biotechnology) according to the instructions.

### Enzyme activity analysis

Liver tissue samples were homogenized in saline, followed by centrifugation (2500 × g, 4°C, 10 min) to obtain the supernatant (*n* = 7). Malondialdehyde (MDA), glutathione peroxidase (GSH-Px) and superoxide dismutase (SOD) in livers were determined with commercially available colorimetric diagnostic kits (Nanjing Jiancheng Bioengineering Institute, China) following the manufacturer’s instructions. The procedures were carried out in duplicate with three parallel samples.

### Untargeted metabolomics

The plasma samples (100 μL) were thawed at 4°C and homogenized in 400 μL of precooled methanol/acetonitrile (1:1, v/v) for 60 s. The untargeted metabolic profiling analysis was conducted by using an ultra-performance liquid chromatography (UPLC) system (1290 Infinity LC, Agilent Technologies, Santa Clara, California, USA) coupled to a quadrupole time-of-flight (TOF) mass spectrometer (Triple TOF 5600, AB SCIEX) with electrospray ionization in positive and negative ionization modes. For chromatographic separation, 2 μL of the extracted sample was injected by an autosampler system at 4°C at a delivery flow rate of 300 μL/min into a liquid chromatography column with a column temperature of 25°C. The mobile phase consisted of A (water + 25 mM ammonium acetate + 25 mM ammonia hydroxide) and B (acetonitrile). The gradient was 95% B and 5% A for 1 min, with a linear reduction to 65% B and 35% A over 13 min, a reduction to 40% B and 60% A over 2 min, maintenance for 2 min and an increase to 95% B and 5% A over 0.1 min, with a 5 min re-equilibration period. Before injection, quality control samples were used to monitor the stability and repeatability of the data produced by the instrument. The screening criteria for differential metabolites were based on a variable importance projection score > 1 and *p* <0.05 (Student’s t-test). The metabolites were analyzed by comparing the molecular ions with compounds in the available biochemical databases, and the pathway analysis of the identified compounds was conducted using the KEGG website (http://www. genome. jp/kegg).

### 16S rRNA gene amplicon sequencing

Intestinal digesta samples were collected after sacrifice. Bacterial DNA was extracted using a QIAamp DNA Stool Kit (Qiagen, Gaithersburg, MD, USA), according to the provided protocols (*n* = 7). The V3-V4 regions of the bacterial 16S rRNA gene was amplified by PCR in triplicate in a 20 μL mixture (2 min at 95°C, followed by 25 cycles of 30 s at 95°C, 30 s at 55°C, 30 s at 72°C and 5 min at 72°C). The primers 314-F (5′-CCTAYGGGRBGCASCAG-′3) and 806-R (5′-GGACTACNNGGGTATCTAAT-′3) were used to target the hypervariable regions. Using the AxyPrep DNA Gel Extraction Kit (Axygen, Union City, CA, USA), the PCR products were purified and then quantified by QuantiFluor^TM^-ST (Promega, Madison, WI, USA). Sequencing libraries were generated using a TruSeq® DNA PCR-Free Sample Preparation Kit. After the library quality was assessed on the Qubit@ 2.0 Fluorometer (Thermo Scientific) and Agilent Bioanalyzer 2100 system, the library was sequenced on an Illumina Novaseq 6000 platform and 250bp paired-end reads were generated. The sequences were merged with FLASH (version 1.2.7) [129] and quality filtered with fastp (version 0.19.6) [130]. Then the high-quality sequences were de-noised using DADA2 [131] plugin in the QIIME2 [132] (version 2020.2) pipeline with recommended parameters to obtain amplicon sequence variants (ASVs). To minimize the effects of sequencing depth, the number of reads from each sample was rarefied to 23834 for comparing all samples at the same sequencing level. The taxonomy of each 16S rRNA sequence was aligned against the bacteria database of Silva version 132 [133]. QIIME2 was also used for the analysis of alpha diversity, including Shannon diversity index, Shannon evenness index, Simpson's diversity index, Simpson's evenness index, Faith's phylogenetic diversity, and beta diversity using the weighted unifrac phylogenetic distance metrics, which was visualized by Non-metric Multidimensional Scaling (NMDS).

### Statistical analysis and visualization

Cladograms of the sow milk bacterial collection were visualized with GraPhlAn version 0.9.7 [134], and the scripts were reused from a previously published paper [135]. SPSS software (version 19.0; IBM Corp., Chicago, IL, USA) was used to evaluate piglet experiment results with one-way analysis of variance and Duncan’s multiple comparison test to determine the statistical significance of the differences among treatment groups. Different letters in a same graph indicate significant statistical differences (*p* < 0.05).

### Accession codes

The accession numbers of 16S rRNA genes of all smBC isolates are listed in **Suppl. Data 1**. The Whole Genome Shotgun project has been deposited at ENA/GenBank under the accession JAEMVT000000000.

## Supplementary Information

**Additional file 1: Figure S1.** The maximum-likelihood tree of the bacterial taxa. The 16S rRNA gene sequences from the isolates were clustered into 271 taxa with a similarity cut-off of 99% using CD-HIT. The closest related species of each taxon are listed next to the taxon numbers of the sow milk microbiota (SMM). Suspected new species are indicated in blue, and the species in the dairy products are indicated in red dots. **Supplementary Data 1.** The 16S rRNA gene sequences and classification of all isolates in smBC.

**Additional file 2: Figure S2.** Light microscopy images of *P. pentosaceus* strains with strong antimicrobial activity under a 63x oil immersion objective. **Supplementary Data 2.** The alignment of 1240 16S rRNA gene sequences against the Silva version 132 16S rRNA gene database, NCBI nucleotide collection (nr/nt) database and DAIRYdb database using BLASTN.

**Additional file 3: Figure S3.** The antimicrobial activity of SMM914. (a) The pH of SMM914 products were determined via test papers. (b) Inhibitory effects of SMM914 against *S. aureus* and enterotoxigenic *E. coli*. The circular wells were filled with different products of SMM914, including MRS medium as the negative control (1&2) and the cell-free supernatant without any treatment (3&4), with 15 min heat inactivation (5&6), with pH adjustment using NaOH (7&8) or with 1 mg/mL protease K treatment (9&10). The substances (1, 3, 5, 7 and 9) were obtained from the fermentation broth after cultivation under anaerobic condition, while others (2, 4, 6, 8 and 10) were obtained from culture under aerobic condition. **Figure S4.** Growth curves and pH value curve of SMM914 after anaerobic or aerobic fermentation. (a) The optical density at 600 nm (OD_600_) was measured from a starting OD_600_ about 0.085. (b) The pH value was recorded with a pH meter. Data are the mean ± s.e.m, *n* = 3. (c) The antimicrobial activities *in vitro* of SMM914 against *S. aureus* and enterotoxigenic *E. coli* at different times of aerobic or anaerobic fermentation.

**Additional file 4: Figure S5.** The annotation and comparison of *P. pentosaceus* SMM914 genome. (a) The distribution of predicted CDSs of *P. pentosaceus* SMM914 in different categories of metabolic function by the online software RAST. (b) A full genome comparison analysis of *P. pentosaceus* SMM914 with other *P. pentosaceus* strains, including *P. pentosaceus* SRCM100194, *P. pentosaceus* GDIAS001, *P. pentosaceus* SL001 and *P. pentosaceus* SRCM102736, visualized by BRIG software. Colors display the percentage of sequence identity based on BLASTN. The two inner rings indicate the GC skew and the GC content. The innermost circle shows the genome coordinates. **Supplementary Data 3.** *P. pentosaceus* SMM914 genes and predicted proteins by Pfam protein database. **Supplementary Table S1.** Oxidative stress resistance genes found in *P. pentosaceus* SMM914. **Supplementary Table S2.** The annotation of antibiotic resistance genes in *P. pentosaceus* SMM914. **Supplementary Table S3.** The annotation of bacterial virulence factors in *P. pentosaceus* SMM914.

**Additional file 5: Figure S6.** Effects of *P. pentosaceus* SMM914 on (a) growth performance, (b) organ relative weight and intestine length in piglets. Data are the mean ± s.e.m. Statistical analysis was conducted by using one-way ANOVA. Data not sharing the same letter in each point were significantly different (*p* < 0.05). **Figure S7.** Effects of *P. pentosaceus* SMM914 on serum biochemical parameters. Data are the mean ± s.e.m (*n* = 7). One-way ANOVA with adjustment for multiple comparisons was conducted. ^a,b^ Within a variable, values with different superscripts differ (*p* < 0.05). **Figure S8.** (a) In HepG2 cell and (b) IPEC-J2 cell, western blotting experiments were to determine the effect of *P. pentosaceus* SMM914 in MRS broth after 24h fermentation on the protein level of Nrf2. (c) The relative changes in protein intensity were analyzed with unpaired Student’s t-test. Different letters in a graph indicate significant statistical differences (*p* < 0.05, *n* = 3).

**Additional file 6: Figure S9.** (a) Alpha diversity comparisons of the gut microbiomes including Ace, Chao, Sobs index, Shannon index, Shannoneven index, Simpson index, Simpsoneven index and Faith's phylogenetic diversity index which were analyzed using a Kruskal-Wallis H test and Tukey-Kramer post hoc test with 95% confidence level. Data are the mean ± s.d (*n* = 7). *ns*, no significant differences. (b) Rarefaction curves for Shannon indices at the genus level in the colon contents. **Figure S10.** (a) Bar charts of relative abundance at the family level in the control and treated groups. (b) Comparison of microbial genera between the high dose group and control. (c) Comparison of microbial families between high dose group and control. Significantly differentially abundant taxa were identified by the Wilcoxon rank-sum test. *p < 0.05; **p < 0.01. **Figure S11.** (a) Linear discriminant analysis score (log10) with a threshold value of 4 from phylum to genus level. (b) Differences in relative abundance of the variable genera in the colonic microbiota among three group. Each data represented the mean and SEM of relative abundance of each genus (*n* = 7). One-way analysis of variance and Duncan’s multiple comparison test to determine the statistical. Different letters in the same graph indicate significant statistical differences (*p* < 0.05).

## Data Availability

The raw sequence data reported in this paper have been deposited (PRJCA003003) in the Genome Sequence Archive in the BIG Data Center, Chinese Academy of Sciences, under accession code CRA002995 for bacterial 16S rRNA gene sequencing data, which are publicly accessible at http://bigd.big.ac.cn/gsa.

## Abbreviations

LAB: Lactic acid bacteria
SMM: Sow milk microbiota
MRS: De Man, Rogosa, and Sharpe
TPY: Trypticase phytone yeast extract
GYP: Glucose yeast extract peptone
CFU: Colony-forming unit
TP: Total protein
ALB: Albumin
ALT: Alanine aminotransferase
LDH: Lactic dehydrogenase
Nrf2: Nuclear factor (erythroid-derived)-like 2
Keap1: Kelch-like ECH-associated protein 1
NQO-1: NADPH quinineoxidoreductase-1
HO-1: Hemeoxygenase-1
CAT: Catalase
MDA: Malondialdehyde
GSH-Px: Glutathione peroxidase
SOD: Superoxide dismutase
DMEM: Dulbecco’s modified Eagle medium
PBS: Phosphate buffer saline
SPI: Sow and Piglet Integration

## References

[1] Stewart CJ (2018). Temporal development of the gut microbiome in early childhood from the TEDDY study. Nature.

[2] Breastfeeding and the Use of Human. Milk. 1997;100(6):1035–9. 10.1542/peds.100.6.1035 9411381

[3] Li Z (2017). Unique Bacteria Community Composition and Co-occurrence in the Milk of Different Ruminants. Sci Rep.

[4] Fitzstevens JL (2017). Systematic Review of the Human Milk Microbiota. Nutr Clin Pract.

[5] Gil-Campos M (2012). Lactobacillus fermentum CECT 5716 is safe and well tolerated in infants of 1-6 months of age: a randomized controlled trial. Pharmacol Res.

[6] Eckert R (2013). Relationships Between Chemical Composition of Colostrum and Milk and Rearing Performance of Piglets During a 21-Day Lactation. Annals of Animal.

[7] Meckel KR, Kiraly DD (2020). Maternal microbes support fetal brain wiring. Nature.

[8] Fernandez L (2013). The human milk microbiota: origin and potential roles in health and disease. Pharmacol Res.

[9] Odamaki T (2018). Genomic diversity and distribution of Bifidobacterium longum subsp. longum across the human lifespan. Sci Rep.

[10] Solieri L, Rutella GS, Tagliazucchi D (2015). Impact of non-starter lactobacilli on release of peptides with angiotensin-converting enzyme inhibitory and antioxidant activities during bovine milk fermentation. Food Microbiol.

[11] Chen T (2020). Functional probiotics of lactic acid bacteria from Hu sheep milk. BMC Microbiol.

[12] Chen W (2018). Lactation Stage-Dependency of the Sow Milk Microbiota. Front Microbiol.

[13] Liu H (2019). Microbial and metabolic alterations in gut microbiota of sows during pregnancy and lactation. FASEB J.

[14] Zuo F, Marcotte H (2021). Advancing mechanistic understanding and bioengineering of probiotic lactobacilli and bifidobacteria by genome editing. Curr Opin Biotechnol.

[15] Papizadeh M (2017). Probiotic characters of Bifidobacterium and Lactobacillus are a result of the ongoing gene acquisition and genome minimization evolutionary trends. Microb Pathog.

[16] Altuntas EG (2014). Purification and mass spectrometry based characterization of a pediocin produced by Pediococcus acidilactici 13. Mol Biol Rep.

[17] Anastasiadou S (2008). Growth and metabolism of a meat isolated strain of Pediococcus pentosaceus in submerged fermentation: Purification, characterization and properties of the produced pediocin SM-1. Enzym Microb Technol.

[18] Porto MC (2017). Pediococcus spp.: An important genus of lactic acid bacteria and pediocin producers. Biotechnol Adv.

[19] Chen W (2012). Comparative Analysis on Antioxidative Ability of Muscle between Laiwu Pig and Large White. Asian-Australas J Anim Sci.

[20] Wang X, et al. Development of Human Breast Milk Microbiota-Associated Mice as a Method to Identify Breast Milk Bacteria Capable of Colonizing Gut. Front Microbiol. 2017;8(1242). 10.3389/fmicb.2017.01242 PMC5504100 28744259

[21] Sellamani M (2016). Antifungal and Zearalenone Inhibitory Activity of Pediococcus pentosaceus Isolated from Dairy Products on Fusarium graminearum. Front Microbiol.

[22] Asami K (2017). Neutralization of Lipopolysaccharide by Heat Shock Protein in Pediococcus pentosaceus AK-23. J Food Sci.

[23] Jiang S (2021). Pediococcus pentosaceus, a future additive or probiotic candidate. Microb Cell Factories.

[24] Yin H (2020). In vitro probiotic properties of Pediococcus pentosaceus L1 and its effects on enterotoxigenic Escherichia coli-induced inflammatory responses in porcine intestinal epithelial cells. Microb Pathog.

[25] Barton MD (2000). Antibiotic use in animal feed and its impact on human healt. Nutr Res Rev.

[26] Lee JW (2015). Multiwall Carbon Nanotube-Induced Apoptosis and Antioxidant Gene Expression in the Gills, Liver, and Intestine of Oryzias latipes. Biomed Res Int.

[27] Moxley RA, et al. Efficacy of Urtoxazumab (TMA-15 Humanized Monoclonal Antibody Specific for Shiga Toxin 2) Against Post-Diarrheal Neurological Sequelae Caused by Escherichia coli O157:H7 Infection in the Neonatal Gnotobiotic Piglet Model. Toxins (Basel). 2017;9(2). 10.3390/toxins9020049 PMC5331429 28134751

[28] Dubreuil JD, Isaacson RE, Schifferli DM (2016). Animal Enterotoxigenic Escherichia coli EcoSal Plus.

[29] Wei H (2008). Fatal infection in human flora-associated piglets caused by the opportunistic pathogen Klebsiella pneumoniae from an apparently healthy human donor. J Vet Med Sci.

[30] Igbinosa IH, Igbinosa EO, Okoh AI (2016). Antibiogram characterization and putative virulence genes in Aeromonas species isolated from pig fecal samples. Environ Sci Pollut Res Int.

[31] Kock R, Cuny C (2020). Multidrug-resistant bacteria in animals and humans. Med Klin Intensivmed Notfmed.

[32] Zheng L, et al. Isolation of swine-derived Lactobacillus plantarum and its synergistic antimicrobial and health-promoting properties with ZnO nanoparticles. J Appl Microbiol. 2020. 10.1111/jam.14605 32027448

[33] De Angelis M (2006). Selection of potential probiotic lactobacilli from pig feces to be used as additives in pelleted feeding. Res Microbiol.

[34] Nair S, Milohanic E, Berche P (2000). ClpC ATPase is required for cell adhesion and invasion of Listeria monocytogenes. Infect Immun.

[35] Gaillot O (2000). The ClpP serine protease is essential for the intracellular parasitism and virulence of Listeria monocytogenes. Mol Microbiol.

[36] Zhang B (2019). Long-term exposure to crotonaldehyde causes heart and kidney dysfunction through induction of inflammatory and oxidative damage in male Wistar rats. Toxicol Mech Methods.

[37] Davis MY (2016). Rapid change of fecal microbiome and disappearance of Clostridium difficile in a colonized infant after transition from breast milk to cow milk. Microbiome.

[38] Leung C (2016). The role of the gut microbiota in NAFLD. Nat Rev Gastroenterol Hepatol.

[39] Napolitano A (2014). Novel Gut-Based Pharmacology of Metformin in Patients with Type 2 Diabetes Mellitus. PLoS One.

[40] Wu W (2018). Dietary sodium butyrate improves intestinal development and function by modulating the microbial community in broilers. PLoS One.

[41] Chen Y (2015). Gut dysbiosis in acute-on-chronic liver failure and its predictive value for mortality. J Gastroenterol Hepatol.

[42] Waters JL, Ley RE (2019). The human gut bacteria Christensenellaceae are widespread, heritable, and associated with health. BMC Biol.

[43] Kong F, et al. Acremonium terricola Culture’s Dose–Response Effects on Lactational Performance, Antioxidant Capacity, and Ruminal Characteristics in Holstein Dairy Cows. Antioxidants. 2022;11(1). 10.3390/antiox11010175 PMC8772898 35052679

[44] Tian H (2020). Effect of Broussonetia papyrifera silage on the serum indicators, hindgut parameters and fecal bacterial community of Holstein heifers. AMB Express.

[45] Buchet A (2017). Effects of age and weaning conditions on blood indicators of oxidative status in pigs. PLoS One.

[46] Smith A (2008). Effect of weaning age on nursery pig and sow reproductive performance. J Swine Health Prod.

[47] Adetoye A (2018). Characterization and anti-salmonella activities of lactic acid bacteria isolated from cattle faeces. BMC Microbiol.

[48] Zheng W (2020). Microbiota-targeted maternal antibodies protect neonates from enteric infection. Nature.

[49] Difilippo E (2016). In Vitro Fermentation of Porcine Milk Oligosaccharides and Galacto-oligosaccharides Using Piglet Fecal Inoculum. J Agric Food Chem.

[50] Fernandez L (2018). Strategies for the Preservation, Restoration and Modulation of the Human Milk Microbiota. Implications for Human Milk Banks and Neonatal Intensive Care Units. Front Microbiol.

[51] Carroll L (1979). Bacteriological criteria for feeding raw breast-milk to babies on neonatal units. Lancet.

[52] West PA, Hewitt JH, Murphy OM (1979). Influence of methods of collection and storage on the bacteriology of human milk. J Appl Bacteriol.

[53] Cacho NT (2017). Personalization of the Microbiota of Donor Human Milk with Mother's Own Milk. Front Microbiol.

[54] Wang L (2019). Natural Products from Mammalian Gut Microbiota. Trends Biotechnol.

[55] Lagier JC (2012). Human gut microbiota: repertoire and variations. Front Cell Infect Microbiol.

[56] Lagier JC (2016). Culture of previously uncultured members of the human gut microbiota by culturomics. Nat Microbiol.

[57] Ramesh V, et al. Comparative evaluation of selected strains of lactobacilli for the development of antioxidant activity in milk. Dairy Sci Technol. 2011;92.

[58] Nie Y (2019). Lactobacillus frumenti improves antioxidant capacity via nitric oxide synthase 1 in intestinal epithelial cells. FASEB J.

[59] Patel PH (2019). Damage sensing by a Nox-Ask1-MKK3-p38 signaling pathway mediates regeneration in the adult Drosophila midgut. Nat Commun.

[60] Darby TM (2019). Lactococcus Lactis Subsp. cremoris Is an Efficacious Beneficial Bacterium that Limits Tissue Injury in the Intestine. iScience.

[61] Mutanen A (2016). Features of liver tissue remodeling in intestinal failure during and after weaning off parenteral nutrition. Surg.

[62] Bernal W (2010). Acute liver failure. Lancet.

[63] Higashikawa F (2016). Antiobesity effect of Pediococcus pentosaceus LP28 on overweight subjects: a randomized, double-blind, placebo-controlled clinical trial. Eur J Clin Nutr.

[64] Yang K (2019). Establishing a method of HPLC involving precolumn derivatization by 2,2'-dithiobis (5-nitropyridine) to determine the sulfites in shrimps in comparison with ion chromatography. Food Sci Nutr.

[65] Sun T (2020). Ultrasound-targeted microbubble destruction optimized HGF-overexpressing bone marrow stem cells to repair fibrotic liver in rats. Stem Cell Res Ther.

[66] Lv L-X, et al. Administration of Lactobacillus salivarius LI01 or Pediococcus pentosaceus LI05 improves acute liver injury induced by D-galactosamine in rats. Appl Microbiol Biotechnol. 2014;98. 10.1007/s00253-014-5638-2 24639205

[67] Kobayashi M (2002). Identification of the interactive interface and phylogenic conservation of the Nrf2-Keap1 system. Genes Cells.

[68] Deng H, Kerppola TK (2013). Regulation of Drosophila metamorphosis by xenobiotic response regulators. PLoS Genet.

[69] Chen-Roetling J, Regan RF (2017). Targeting the Nrf2-Heme Oxygenase-1 Axis after Intracerebral Hemorrhage. Curr Pharm Des.

[70] Hao L (2021). Pediococcus pentosaceus ZJUAF-4 relieves oxidative stress and restores the gut microbiota in diquat-induced intestinal injury. Appl Microbiol Biotechnol.

[71] Saeedi BJ (2020). Gut-Resident Lactobacilli Activate Hepatic Nrf2 and Protect Against Oxidative Liver Injury. Cell Metab.

[72] Schwab CG (1992). Amino acid limitation and flow to the duodenum at four stages of lactation. 2. Extent of lysine limitation. J Dairy Sci.

[73] Zeitz JO (2019). Effects of L-methionine on performance, gut morphology and antioxidant status in gut and liver of piglets in relation to DL-methionine. J Anim Physiol Anim Nutr (Berl).

[74] Couturier J (2012). Glutathione- and glutaredoxin-dependent reduction of methionine sulfoxide reductase A. FEBS Lett.

[75] Sistla S, Rao DN (2004). S-Adenosyl-L-methionine-dependent restriction enzymes. Crit Rev Biochem Mol Biol.

[76] Lin LC, Hsu JH, Wang LC (2010). Identification of novel inhibitors of 1-aminocyclopropane-1-carboxylic acid synthase by chemical screening in Arabidopsis thaliana. J Biol Chem.

[77] Riedijk MA (2007). Methionine transmethylation and transsulfuration in the piglet gastrointestinal tract. Proc Natl Acad Sci U S A.

[78] Zhou YF (2018). Methionine and choline supply alter transmethylation, transsulfuration, and cytidine 5'-diphosphocholine pathways to different extents in isolated primary liver cells from dairy cows. J Dairy Sci.

[79] Swennen Q (2011). Effects of dietary protein content and 2-hydroxy-4-methylthiobutanoic acid or DL-methionine supplementation on performance and oxidative status of broiler chickens. Br J Nutr.

[80] Wu G (2004). Glutathione metabolism and its implications for health. J Nutr.

[81] Bos C (2005). Postprandial intestinal and whole body nitrogen kinetics and distribution in piglets fed a single meal. Am J Physiol Endocrinol Metab.

[82] Jiao N (2015). L-Glutamate Enhances Barrier and Antioxidative Functions in Intestinal Porcine Epithelial Cells. J Nutr.

[83] Bachhawat AK, Yadav S (2018). The glutathione cycle: Glutathione metabolism beyond the gamma-glutamyl cycle. IUBMB Life.

[84] Gamarra Y (2019). Pyroglutamic acidosis by glutathione regeneration blockage in critical patients with septic shock. Crit Care.

[85] Zeisel SH (2012). Dietary choline deficiency causes DNA strand breaks and alters epigenetic marks on DNA and histones. Mutat Res.

[86] Guo WX (2004). Reactive oxygen species in choline deficiency-induced apoptosis in rat hepatocytes. Free Radic Biol Med.

[87] Tabassum S (2017). Chronic choline supplementation improves cognitive and motor performance via modulating oxidative and neurochemical status in rats. Pharmacol Biochem Behav.

[88] Zhou Z, et al. Methionine and Choline Supply during the Periparturient Period Alter Plasma Amino Acid and One-Carbon Metabolism Profiles to Various Extents: Potential Role in Hepatic Metabolism and Antioxidant Status. Nutrients. 2016;9(1). 10.3390/nu9010010 PMC5295054 28036059

[89] Li B (2015). Effects of Choline on Meat Quality and Intramuscular Fat in Intrauterine Growth Retardation Pigs. PLoS One.

[90] Wu P (2017). A Comparative Study on Antioxidant System in Fish Hepatopancreas and Intestine Affected by Choline Deficiency: Different Change Patterns of Varied Antioxidant Enzyme Genes and Nrf2 Signaling Factors. PLoS One.

[91] Coleman DN (2019). Hepatic betaine-homocysteine methyltransferase and methionine synthase activity and intermediates of the methionine cycle are altered by choline supply during negative energy balance in Holstein cows. J Dairy Sci.

[92] Jones B, et al. Activation of proline biosynthesis is critical to maintain glutamate homeostasis during acute methamphetamine exposure. 2021;11:1. 10.1038/s41598-020-80917-7 PMC7809342 33446840

[93] Ortiz JG, Cordero ML, Rosado A (1997). Proline-glutamate interactions in the CNS. Prog Neuro-Psychopharmacol Biol Psychiatry.

[94] Ball RO, Atkinson JL, Bayley HS (1986). Proline as an essential amino acid for the young pig. Br J Nutr.

[95] Wang J (2015). Oral administration of putrescine and proline during the suckling period improves epithelial restitution after early weaning in piglets. J Anim Sci.

[96] O'Mahony SM (2011). Maternal separation as a model of brain-gut axis dysfunction. Psychopharmacology.

[97] Kember RL (2012). Maternal separation is associated with strain-specific responses to stress and epigenetic alterations to Nr3c1, Avp, and Nr4a1 in mouse. Brain Behav.

[98] Becker BA (1985). Peripheral concentrations of cortisol as an indicator of stress in the pig. Am J Vet Res.

[99] Du X, Pang TY (2015). Is Dysregulation of the HPA-Axis a Core Pathophysiology Mediating Co-Morbid Depression in Neurodegenerative Diseases?. Front Psychiatry.

[100] Kulak A (2013). Redox dysregulation in the pathophysiology of schizophrenia and bipolar disorder: insights from animal models. Antioxid Redox Signal.

[101] Lopes IS (2018). Riparin II ameliorates corticosterone-induced depressive-like behavior in mice: Role of antioxidant and neurotrophic mechanisms. Neurochem Int.

[102] Yamada S (2017). Cholic Acid Enhances Visceral Adiposity, Atherosclerosis and Nonalcoholic Fatty Liver Disease in Microminipigs. J Atheroscler Thromb.

[103] Carrier A (2017). Metabolic Syndrome and Oxidative Stress: A Complex Relationship. Antioxid Redox Signal.

[104] Sommerfeld A, Reinehr R, Haussinger D (2009). Bile acid-induced epidermal growth factor receptor activation in quiescent rat hepatic stellate cells can trigger both proliferation and apoptosis. J Biol Chem.

[105] Lin X (2018). Lactobacillus plantarum AR501 Alleviates the Oxidative Stress of D-Galactose-Induced Aging Mice Liver by Upregulation of Nrf2-Mediated Antioxidant Enzyme Expression. J Food Sci.

[106] Jiang T (2019). Chitosan Oligosaccharides Show Protective Effects in Coronary Heart Disease by Improving Antioxidant Capacity via the Increase in Intestinal Probiotics. Oxidative Med Cell Longev.

[107] Wu CH (2019). D-methionine alleviates cisplatin-induced mucositis by restoring the gut microbiota structure and improving intestinal inflammation. Ther Adv Med Oncol.

[108] Hu R (2019). Fermented carrot juice attenuates type 2 diabetes by mediating gut microbiota in rats. Food Funct.

[109] Xu HJ, et al. Growth performance, digestibility, blood metabolites, ruminal fermentation, and bacterial communities in response to the inclusion of gallic acid in the starter feed of preweaning dairy calves. J Dairy Sci. 2022. 10.3168/jds.2021-20838 35086717

[110] Wang K (2018). Propolis from Different Geographic Origins Decreases Intestinal Inflammation and Bacteroides spp. Populations in a Model of DSS-Induced Colitis. Mol Nutr Food Res.

[111] Takano S, et al. Density-Dependent Recycling Promotes the Long-Term Survival of Bacterial Populations during Periods of Starvation. mBio. 2017;8(1). 10.1128/mBio.02336-16 PMC5296608 28174316

[112] Wang D (2016). Isolation and Identification of Lactic Acid Bacteria from Traditional Dairy Products in Baotou and Bayannur of Midwestern Inner Mongolia and q-PCR Analysis of Predominant Species. Korean J Food Sci Anim Resour.

[113] Grill JP (2000). Effects of Lactobacillus amylovorus and Bifidobacterium breve on cholesterol. Lett Appl Microbiol.

[114] Limkhuansuwan V, Chaiprasert P (2010). Decolorization of molasses melanoidins and palm oil mill effluent phenolic compounds by fermentative lactic acid bacteria. J Environ Sci (China).

[115] Hu J (2018). A Microbiota-Derived Bacteriocin Targets the Host to Confer Diarrhea Resistance in Early-Weaned Piglets. Cell Host Microbe.

[116] Janda JM, Abbott SL (2007). 16S rRNA gene sequencing for bacterial identification in the diagnostic laboratory: pluses, perils, and pitfalls. J Clin Microbiol.

[117] Li R (2010). De novo assembly of human genomes with massively parallel short read sequencing. Genome Res.

[118] Li R (2008). SOAP: short oligonucleotide alignment program. Bioinformatics.

[119] Bankevich A (2012). SPAdes: a new genome assembly algorithm and its applications to single-cell sequencing. J Comput Biol.

[120] Simpson JT (2009). ABySS: a parallel assembler for short read sequence data. Genome Res.

[121] Lin SH, Liao YC (2013). CISA: contig integrator for sequence assembly of bacterial genomes. PLoS One.

[122] Saha S (2008). Empirical comparison of ab initio repeat finding programs. Nucleic Acids Res.

[123] Besemer J, Lomsadze A, Borodovsky M (2001). GeneMarkS: a self-training method for prediction of gene starts in microbial genomes. Implications for finding sequence motifs in regulatory regions. Nucleic Acids Res.

[124] Cantarel BL (2009). The Carbohydrate-Active EnZymes database (CAZy): an expert resource for Glycogenomics. Nucleic Acids Res.

[125] Punta M (2012). The Pfam protein families database. Nucleic Acids Res.

[126] Chen L (2005). VFDB: a reference database for bacterial virulence factors. Nucleic Acids Res.

[127] McArthur AG (2013). The comprehensive antibiotic resistance database. Antimicrob Agents Chemother.

[128] Ponce AG (2008). Preliminary characterization of bacteriocin-like substances from lactic acid bacteria isolated from organic leafy vegetables. LWT Food Sci Technol.

[129] Magoč T, Salzberg SL (2011). FLASH: fast length adjustment of short reads to improve genome assemblies. Bioinformatics.

[130] Chen S (2018). fastp: an ultra-fast all-in-one FASTQ preprocessor. Bioinformatics.

[131] Callahan BJ (2016). DADA2: High-resolution sample inference from Illumina amplicon data. Nat Methods.

[132] Bolyen E (2019). Reproducible, interactive, scalable and extensible microbiome data science using QIIME 2. Nat Biotechnol.

[133] Quast C (2013). The SILVA ribosomal RNA gene database project: improved data processing and web-based tools. Nucleic Acids Res.

[134] Asnicar F (2015). Compact graphical representation of phylogenetic data and metadata with GraPhlAn. PeerJ.

[135] Zhang J (2019). NRT1.1B is associated with root microbiota composition and nitrogen use in field-grown rice. Nat Biotechnol.

