# Supplementary figures and images for: Antioxidant potential of Pediococcus pentosaceus strains from the sow milk bacterial collection in weaned piglets
Source: Microbiome. 2022 Jun 1;10:83. doi: 10.1186/s40168-022-01278-z (PMC9158380; doi:10.1186/s40168-022-01278-z)

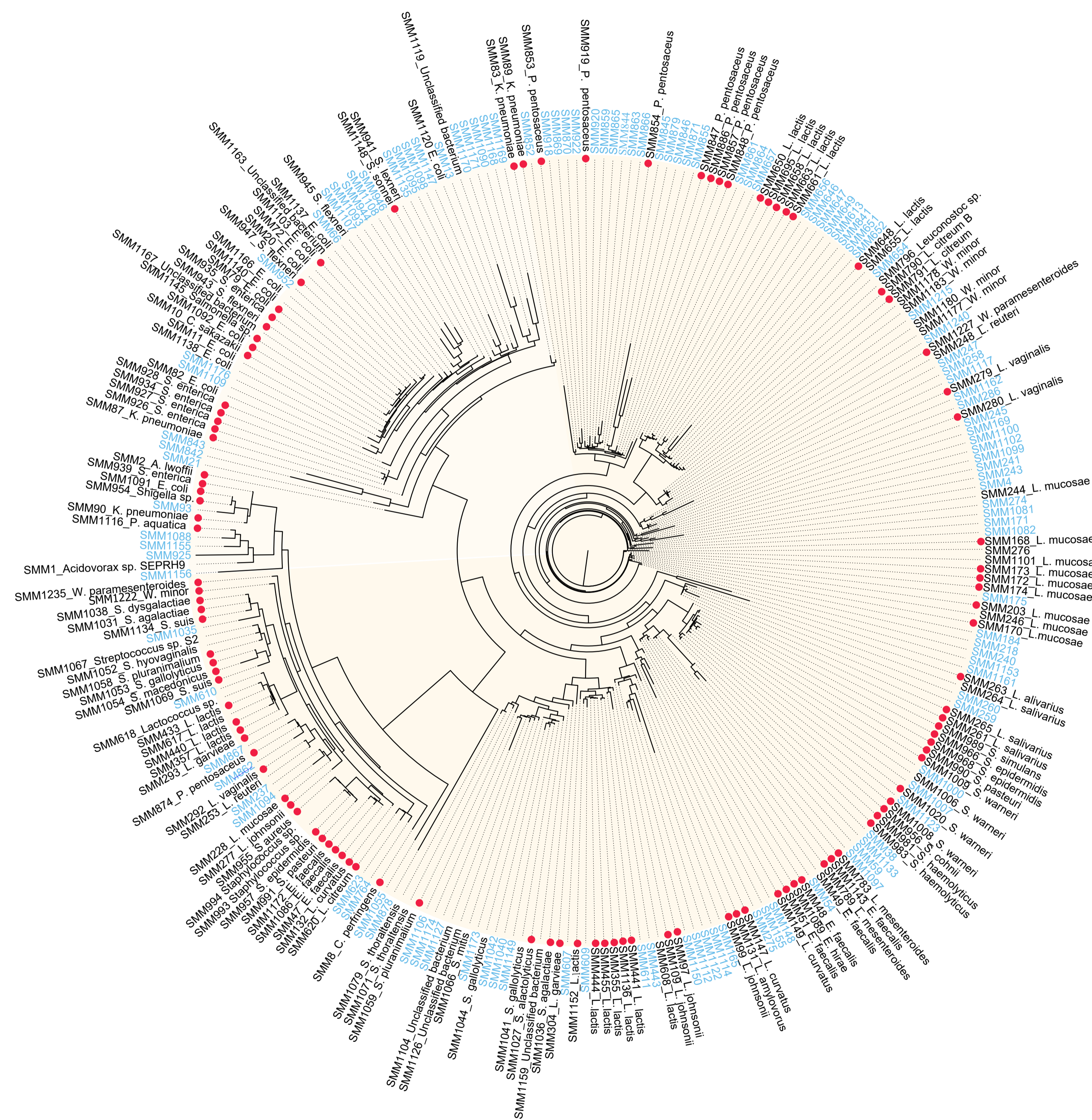

Supplement: Supplementary file 2 — Additional file 1: Figure S1. The maximum-likelihood tree of the bacterial taxa. The 16S rRNA gene sequences from the isolates were clustered into 271 taxa with a similarity cut-off of 99% using CD-HIT. The closest related species of each taxon are listed next to the taxon numbers of the sow milk microbiota (SMM). Suspected new species are indicated in blue, and the species in the dairy products are indicated in red dots. Supplementary Data 1. The 16S rRNA gene sequences and classification of all isolates in smBC. [file 40168_2022_1278_MOESM1_ESM.zip › Suppl. Fig. S1.pdf]

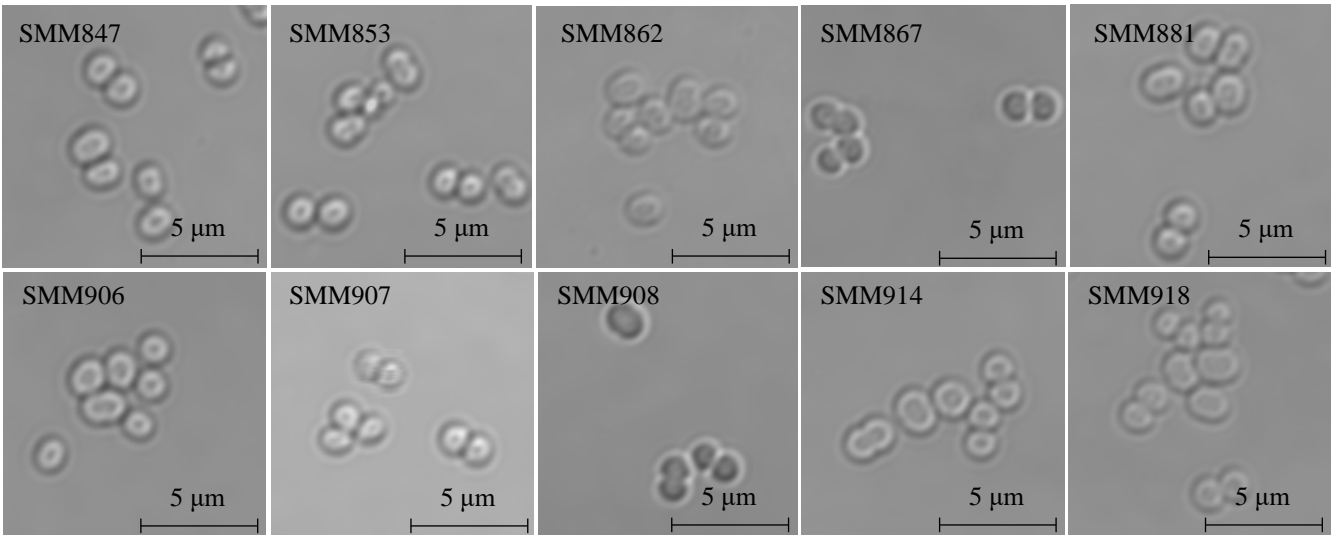

Suppl. Fig. S2

Supplement: Supplementary file 3 — Additional file 2: Figure S2. Light microscopy images of P. pentosaceus strains with strong antimicrobial activity under a 63x oil immersion objective. Supplementary Data 2. The alignment of 1240 16S rRNA gene sequences against the Silva version 132 16S rRNA gene database, NCBI nucleotide collection (nr/nt) database and DAIRYdb database using BLASTN. [file 40168_2022_1278_MOESM2_ESM.zip › Suppl. Fig. S2.pdf]

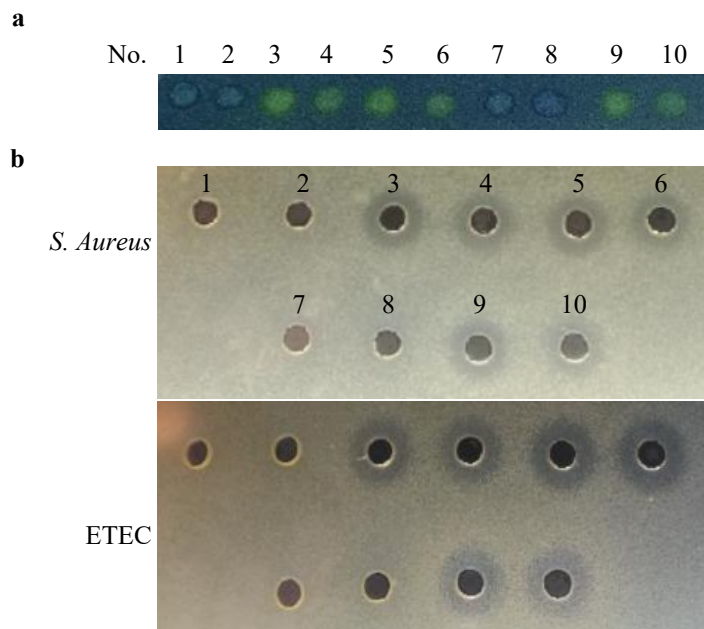

Suppl. Fig. S3

Supplement: Supplementary file 4 — Additional file 3: Figure S3. The antimicrobial activity of SMM914. (a) The pH of SMM914 products were determined via test papers. (b) Inhibitory effects of SMM914 against S. aureus and enterotoxigenic E. coli. The circular wells were filled with different products of SMM914, including MRS medium as the negative control (1&2) and the cell-free supernatant without any treatment (3&4), with 15 min heat inactivation (5&6), with pH adjustment using NaOH (7&8) or with 1 mg/mL protease K treatment (9&10). The substances (1, 3, 5, 7 and 9) were obtained from the fermentation broth after cultivation under anaerobic condition, while others (2, 4, 6, 8 and 10) were obtained from culture under aerobic condition. Figure S4. Growth curves and pH value curve of SMM914 after anaerobic or aerobic fermentation. (a) The optical density at 600 nm (OD600) was measured from a starting OD600 about 0.085. (b) The pH value was recorded with a pH meter. Data are the mean ± s.e.m, n = 3. (c) The antimicrobial activities in vitro of SMM914 against S. aureus and enterotoxigenic E. coli at different times of aerobic or anaerobic fermentation. [file 40168_2022_1278_MOESM3_ESM.zip › Suppl. Fig. S3.pdf]

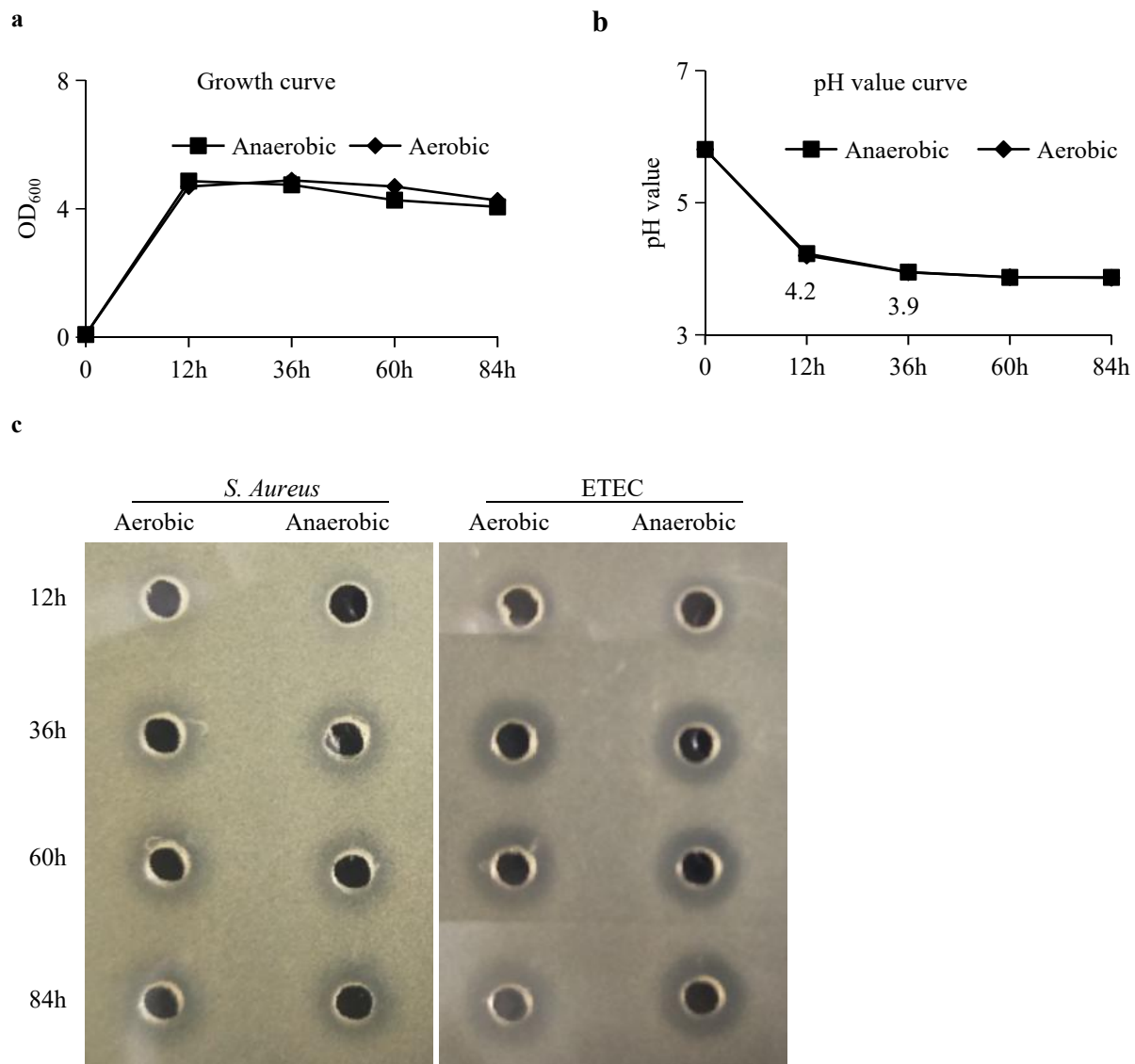

Suppl. Fig. S4

Supplement: Supplementary file 4 — Additional file 3: Figure S3. The antimicrobial activity of SMM914. (a) The pH of SMM914 products were determined via test papers. (b) Inhibitory effects of SMM914 against S. aureus and enterotoxigenic E. coli. The circular wells were filled with different products of SMM914, including MRS medium as the negative control (1&2) and the cell-free supernatant without any treatment (3&4), with 15 min heat inactivation (5&6), with pH adjustment using NaOH (7&8) or with 1 mg/mL protease K treatment (9&10). The substances (1, 3, 5, 7 and 9) were obtained from the fermentation broth after cultivation under anaerobic condition, while others (2, 4, 6, 8 and 10) were obtained from culture under aerobic condition. Figure S4. Growth curves and pH value curve of SMM914 after anaerobic or aerobic fermentation. (a) The optical density at 600 nm (OD600) was measured from a starting OD600 about 0.085. (b) The pH value was recorded with a pH meter. Data are the mean ± s.e.m, n = 3. (c) The antimicrobial activities in vitro of SMM914 against S. aureus and enterotoxigenic E. coli at different times of aerobic or anaerobic fermentation. [file 40168_2022_1278_MOESM3_ESM.zip › Suppl. Fig. S4.pdf]

a

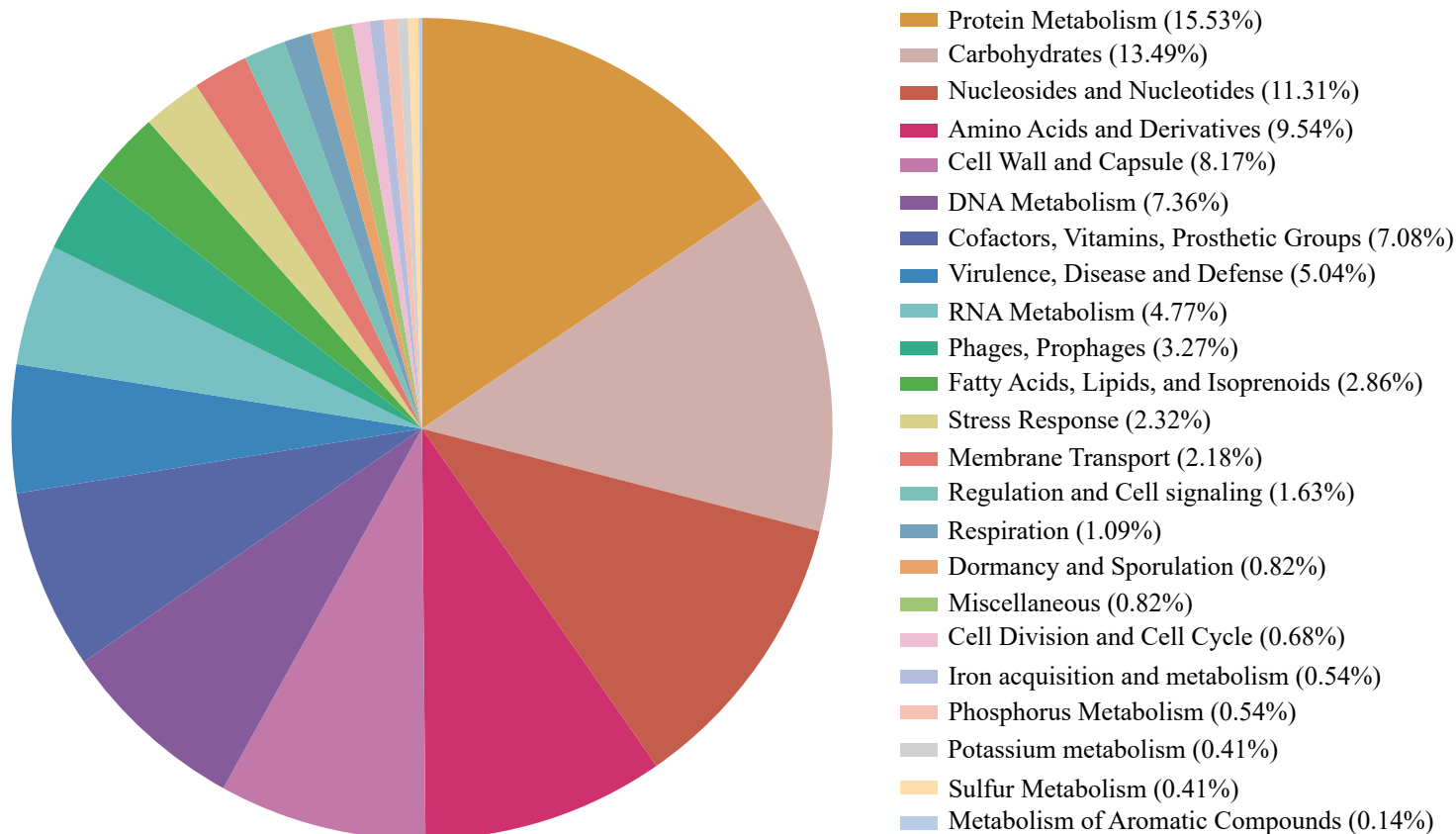

b

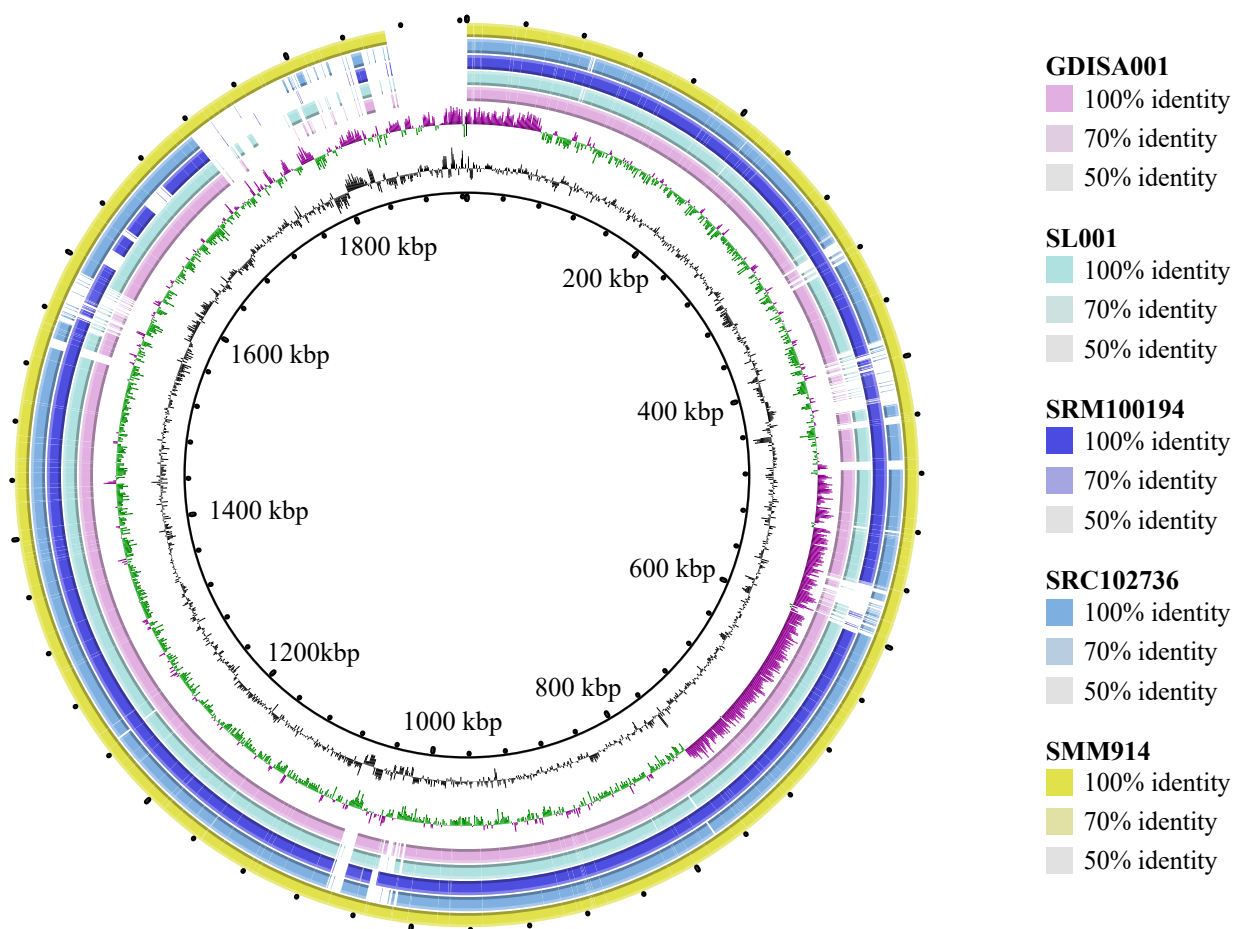

Supplement: Supplementary file 5 — Additional file 4: Figure S5. The annotation and comparison of P. pentosaceus SMM914 genome. (a) The distribution of predicted CDSs of P. pentosaceus SMM914 in different categories of metabolic function by the online software RAST. (b) A full genome comparison analysis of P. pentosaceus SMM914 with other P. pentosaceus strains, including P. pentosaceus SRCM100194, P. pentosaceus GDIAS001, P. pentosaceus SL001 and P. pentosaceus SRCM102736, visualized by BRIG software. Colors display the percentage of sequence identity based on BLASTN. The two inner rings indicate the GC skew and the GC content. The innermost circle shows the genome coordinates. Supplementary Data 3. P. pentosaceus SMM914 genes and predicted proteins by Pfam protein database. Supplementary Table S1. Oxidative stress resistance genes found in P. pentosaceus SMM914. Supplementary Table S2. The annotation of antibiotic resistance genes in P. pentosaceus SMM914. Supplementary Table S3. The annotation of bacterial virulence factors in P. pentosaceus SMM914. [file 40168_2022_1278_MOESM4_ESM.zip › Suppl. Fig. S5.pdf]

**a**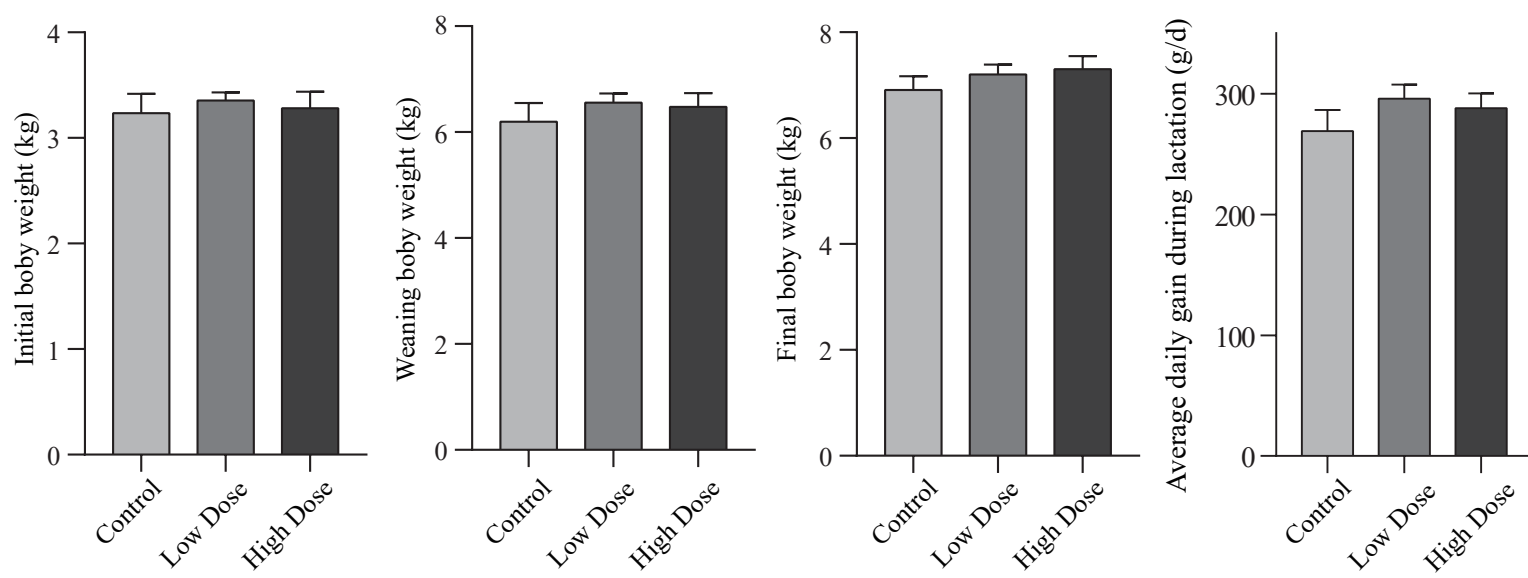**b**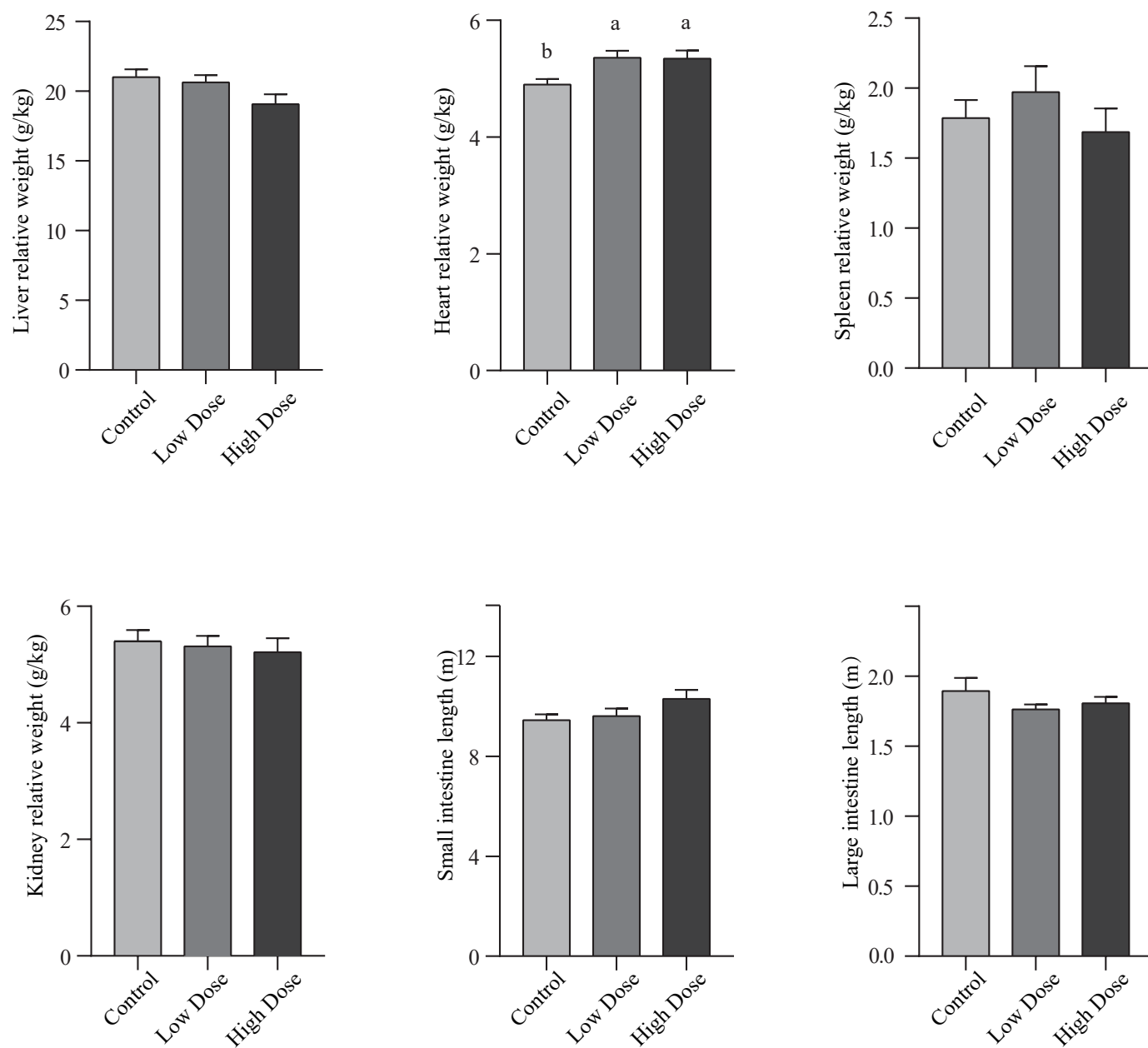

Suppl. Fig. S6

Supplement: Supplementary file 6 — Additional file 5: Figure S6. Effects of P. pentosaceus SMM914 on (a) growth performance, (b) organ relative weight and intestine length in piglets. Data are the mean ± s.e.m. Statistical analysis was conducted by using one-way ANOVA. Data not sharing the same letter in each point were significantly different (p < 0.05). Figure S7. Effects of P. pentosaceus SMM914 on serum biochemical parameters. Data are the mean ± s.e.m (n = 7). One-way ANOVA with adjustment for multiple comparisons was conducted. a,b Within a variable, values with different superscripts differ (p < 0.05). Figure S8. (a) In HepG2 cell and (b) IPEC-J2 cell, western blotting experiments were to determine the effect of P. pentosaceus SMM914 in MRS broth after 24h fermentation on the protein level of Nrf2. (c) The relative changes in protein intensity were analyzed with unpaired Student’s t-test. Different letters in a graph indicate significant statistical differences (p < 0.05, n = 3). [file 40168_2022_1278_MOESM5_ESM.zip › Suppl. Fig. S6.pdf]

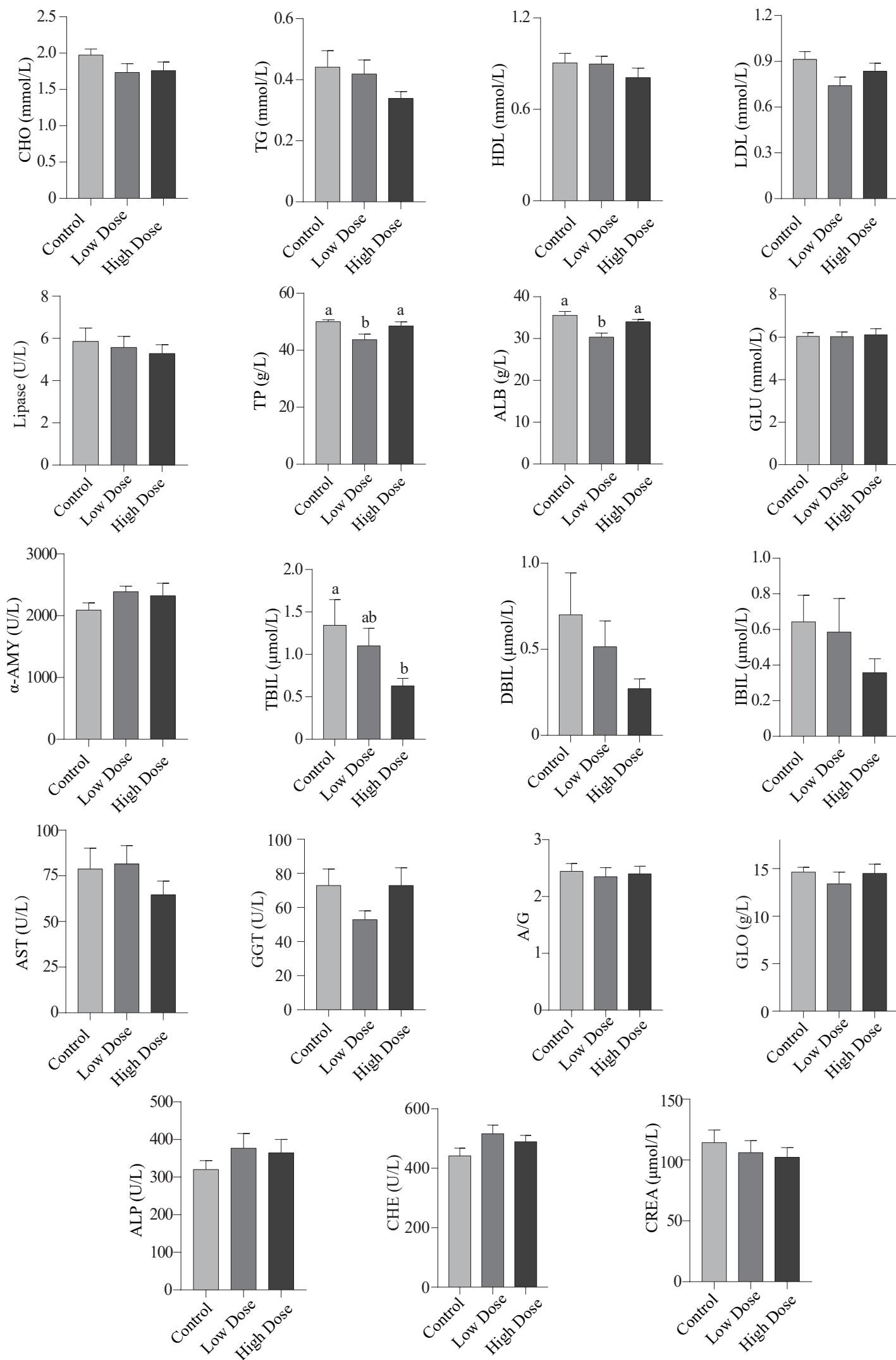

Suppl. Fig. S7

Supplement: Supplementary file 6 — Additional file 5: Figure S6. Effects of P. pentosaceus SMM914 on (a) growth performance, (b) organ relative weight and intestine length in piglets. Data are the mean ± s.e.m. Statistical analysis was conducted by using one-way ANOVA. Data not sharing the same letter in each point were significantly different (p < 0.05). Figure S7. Effects of P. pentosaceus SMM914 on serum biochemical parameters. Data are the mean ± s.e.m (n = 7). One-way ANOVA with adjustment for multiple comparisons was conducted. a,b Within a variable, values with different superscripts differ (p < 0.05). Figure S8. (a) In HepG2 cell and (b) IPEC-J2 cell, western blotting experiments were to determine the effect of P. pentosaceus SMM914 in MRS broth after 24h fermentation on the protein level of Nrf2. (c) The relative changes in protein intensity were analyzed with unpaired Student’s t-test. Different letters in a graph indicate significant statistical differences (p < 0.05, n = 3). [file 40168_2022_1278_MOESM5_ESM.zip › Suppl. Fig. S7.pdf]

**a**

HepG2 cell

MRS

SMM914

Nrf2

Histone H3

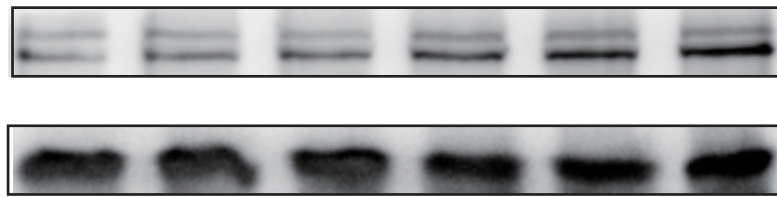**b**

IPEC-J2 cell

MRS

SMM914

Nrf2

PCNA

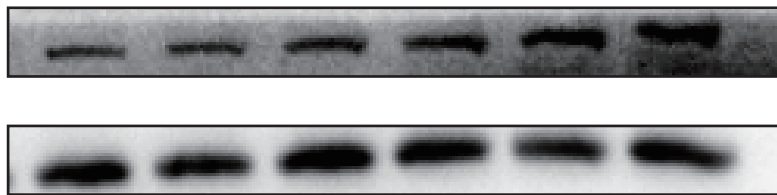**c**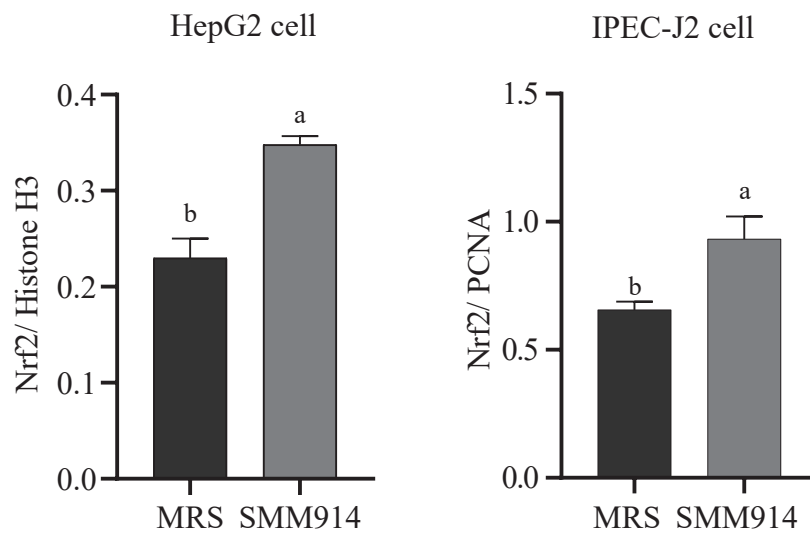

Suppl. Fig. S8

Supplement: Supplementary file 6 — Additional file 5: Figure S6. Effects of P. pentosaceus SMM914 on (a) growth performance, (b) organ relative weight and intestine length in piglets. Data are the mean ± s.e.m. Statistical analysis was conducted by using one-way ANOVA. Data not sharing the same letter in each point were significantly different (p < 0.05). Figure S7. Effects of P. pentosaceus SMM914 on serum biochemical parameters. Data are the mean ± s.e.m (n = 7). One-way ANOVA with adjustment for multiple comparisons was conducted. a,b Within a variable, values with different superscripts differ (p < 0.05). Figure S8. (a) In HepG2 cell and (b) IPEC-J2 cell, western blotting experiments were to determine the effect of P. pentosaceus SMM914 in MRS broth after 24h fermentation on the protein level of Nrf2. (c) The relative changes in protein intensity were analyzed with unpaired Student’s t-test. Different letters in a graph indicate significant statistical differences (p < 0.05, n = 3). [file 40168_2022_1278_MOESM5_ESM.zip › Suppl. Fig. S8.pdf]

a

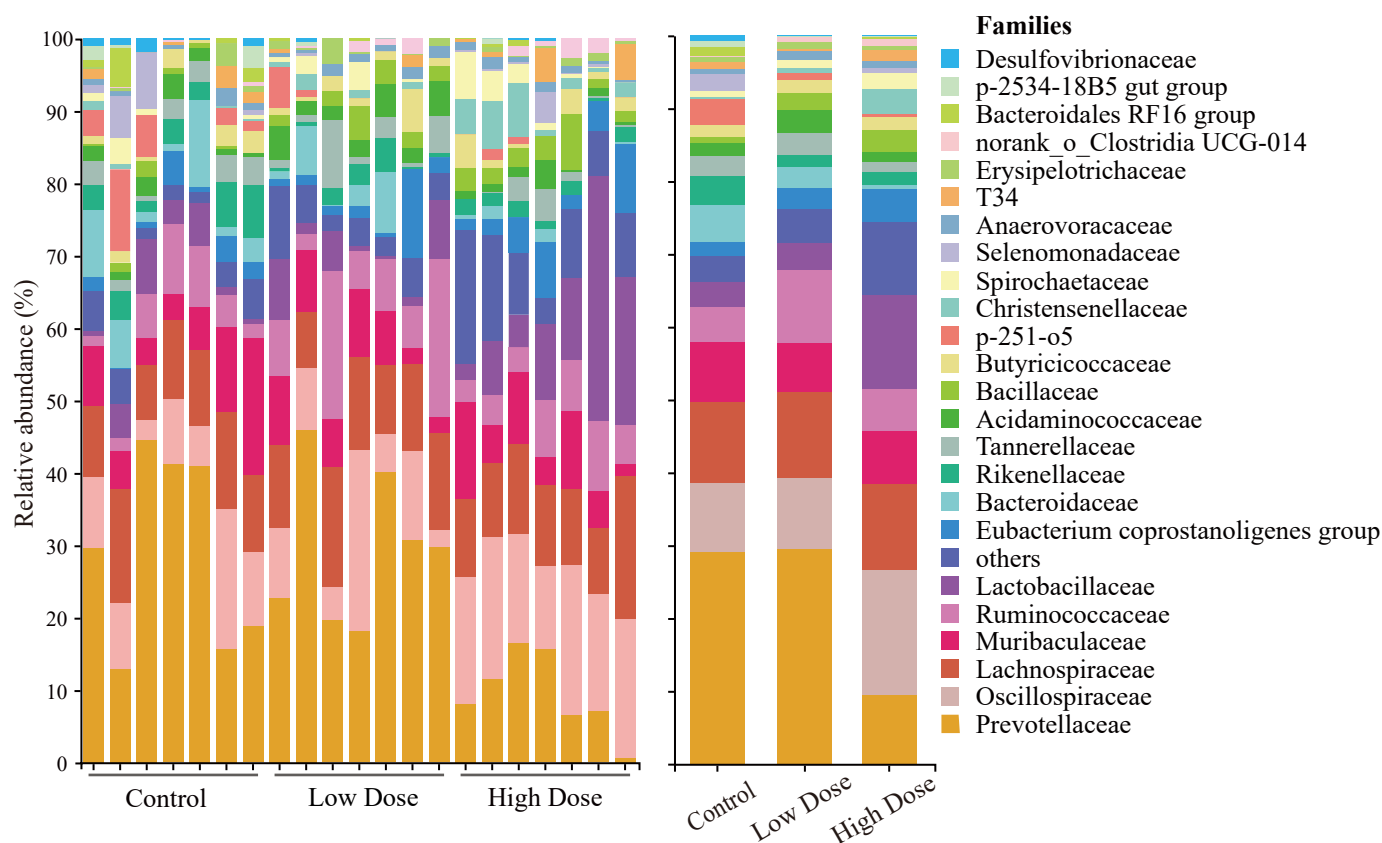

b

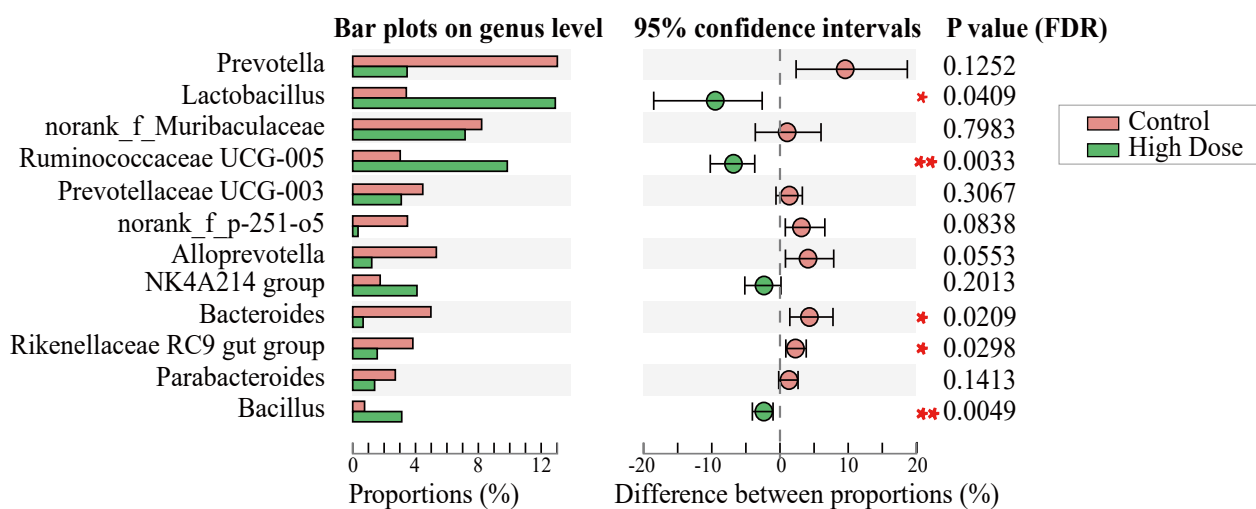

c

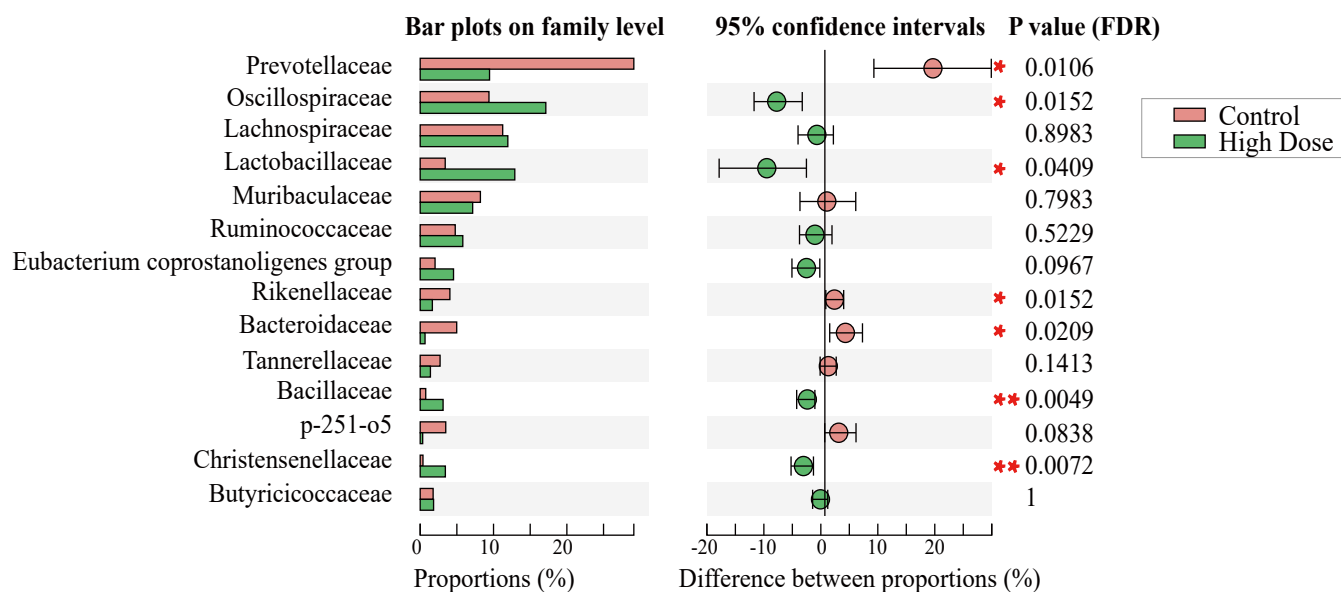

Supplement: Supplementary file 7 — Additional file 6: Figure S9. (a) Alpha diversity comparisons of the gut microbiomes including Ace, Chao, Sobs index, Shannon index, Shannoneven index, Simpson index, Simpsoneven index and Faith's phylogenetic diversity index which were analyzed using a Kruskal-Wallis H test and Tukey-Kramer post hoc test with 95% confidence level. Data are the mean ± s.d (n = 7). ns, no significant differences. (b) Rarefaction curves for Shannon indices at the genus level in the colon contents. Figure S10. (a) Bar charts of relative abundance at the family level in the control and treated groups. (b) Comparison of microbial genera between the high dose group and control. (c) Comparison of microbial families between high dose group and control. Significantly differentially abundant taxa were identified by the Wilcoxon rank-sum test. *p < 0.05; **p < 0.01. Figure S11. (a) Linear discriminant analysis score (log10) with a threshold value of 4 from phylum to genus level. (b) Differences in relative abundance of the variable genera in the colonic microbiota among three group. Each data represented the mean and SEM of relative abundance of each genus (n = 7). One-way analysis of variance and Duncan’s multiple comparison test to determine the statistical. Different letters in the same graph indicate significant statistical differences (p < 0.05). [file 40168_2022_1278_MOESM6_ESM.zip › Suppl. Fig. S10.pdf]

a

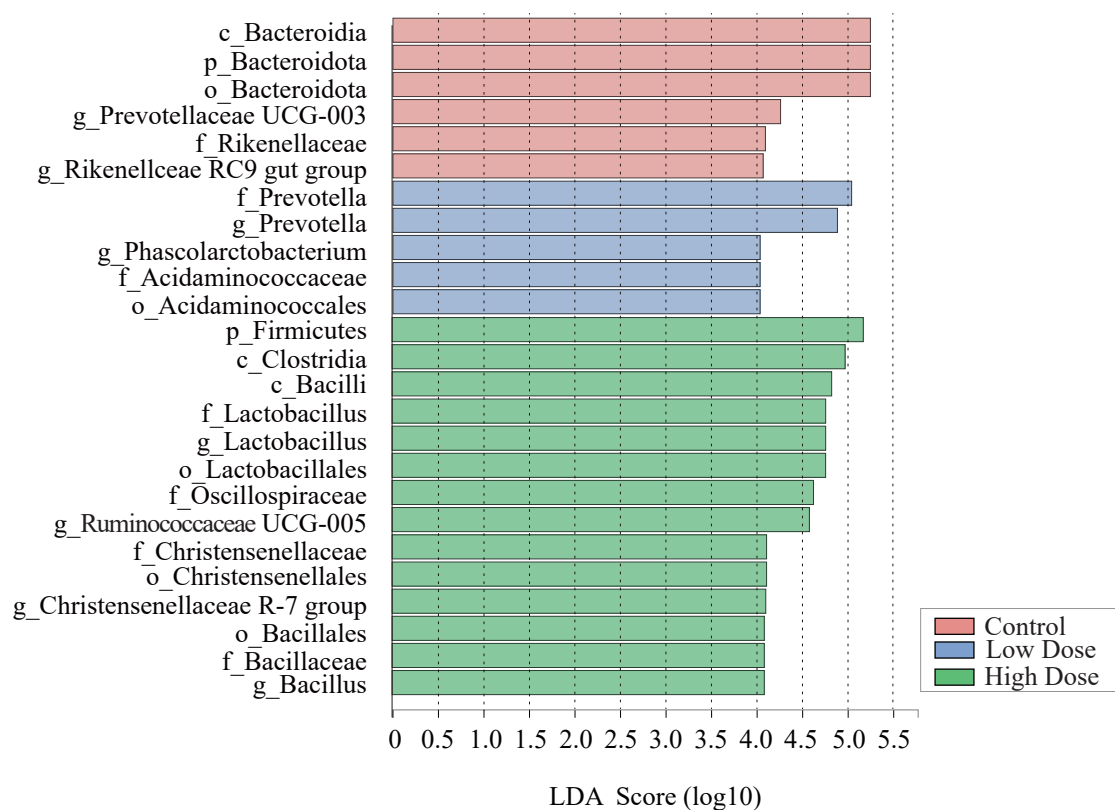

b

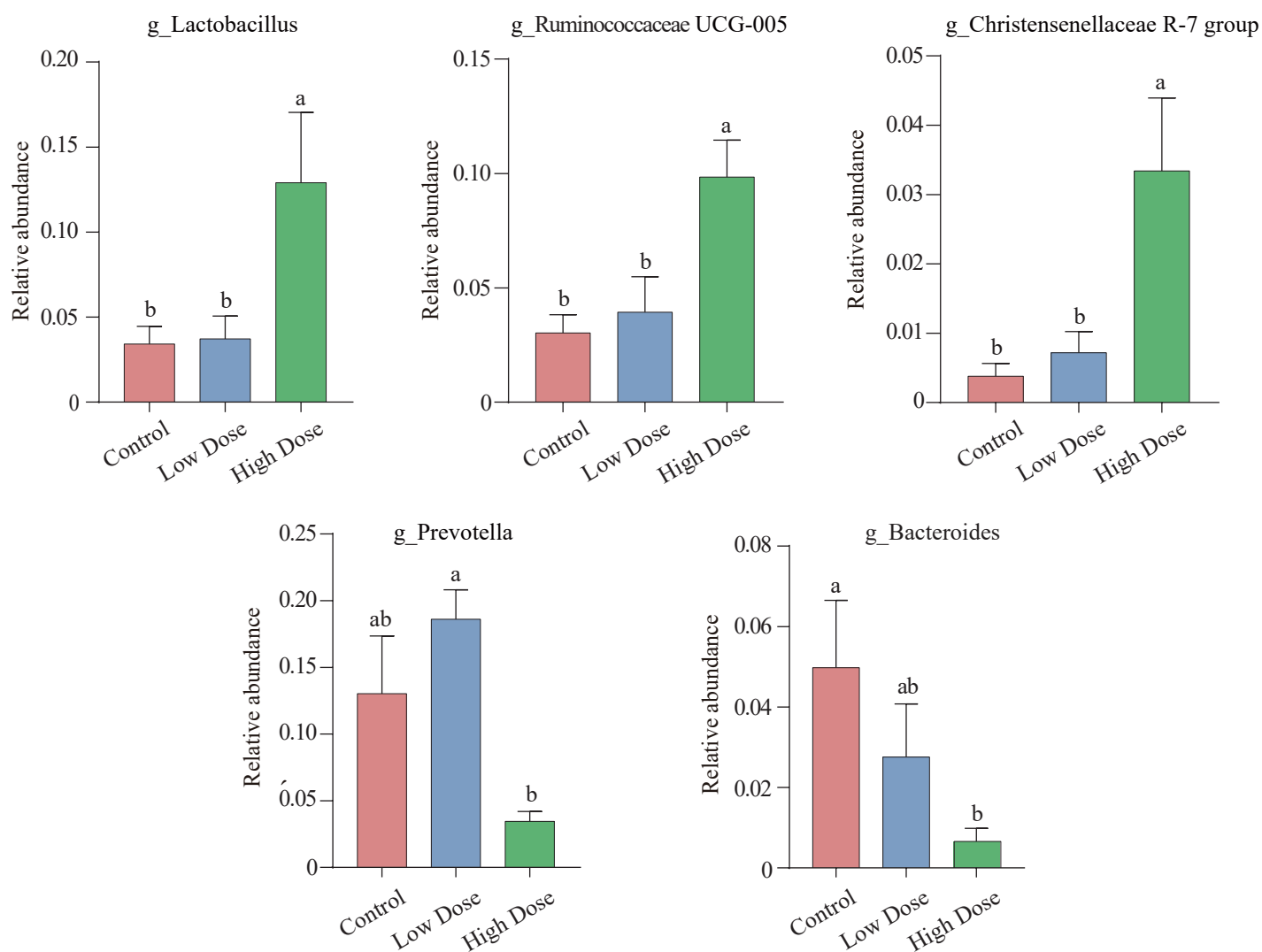

Supplement: Supplementary file 7 — Additional file 6: Figure S9. (a) Alpha diversity comparisons of the gut microbiomes including Ace, Chao, Sobs index, Shannon index, Shannoneven index, Simpson index, Simpsoneven index and Faith's phylogenetic diversity index which were analyzed using a Kruskal-Wallis H test and Tukey-Kramer post hoc test with 95% confidence level. Data are the mean ± s.d (n = 7). ns, no significant differences. (b) Rarefaction curves for Shannon indices at the genus level in the colon contents. Figure S10. (a) Bar charts of relative abundance at the family level in the control and treated groups. (b) Comparison of microbial genera between the high dose group and control. (c) Comparison of microbial families between high dose group and control. Significantly differentially abundant taxa were identified by the Wilcoxon rank-sum test. *p < 0.05; **p < 0.01. Figure S11. (a) Linear discriminant analysis score (log10) with a threshold value of 4 from phylum to genus level. (b) Differences in relative abundance of the variable genera in the colonic microbiota among three group. Each data represented the mean and SEM of relative abundance of each genus (n = 7). One-way analysis of variance and Duncan’s multiple comparison test to determine the statistical. Different letters in the same graph indicate significant statistical differences (p < 0.05). [file 40168_2022_1278_MOESM6_ESM.zip › Suppl. Fig. S11.pdf]

**a**

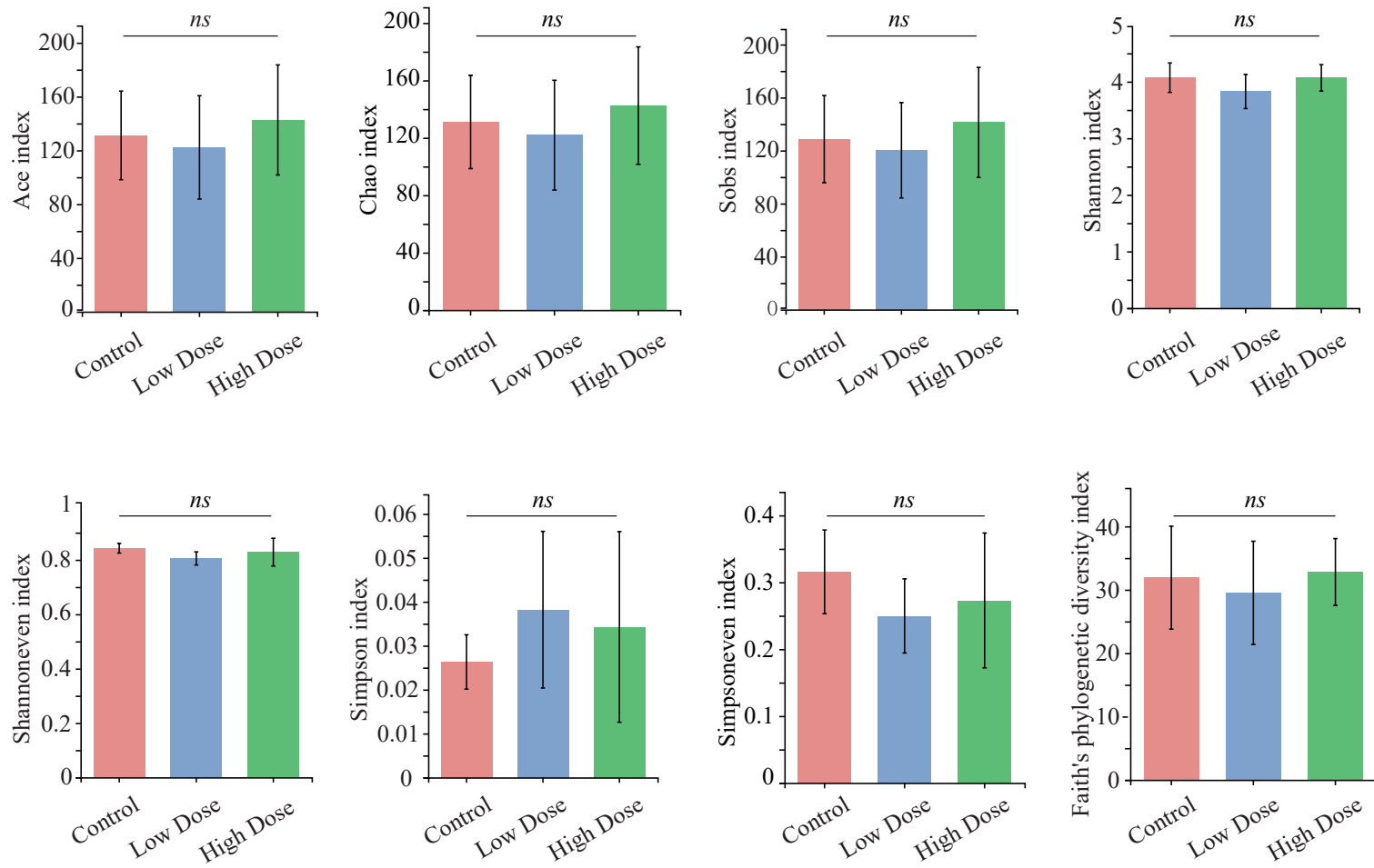

**b**

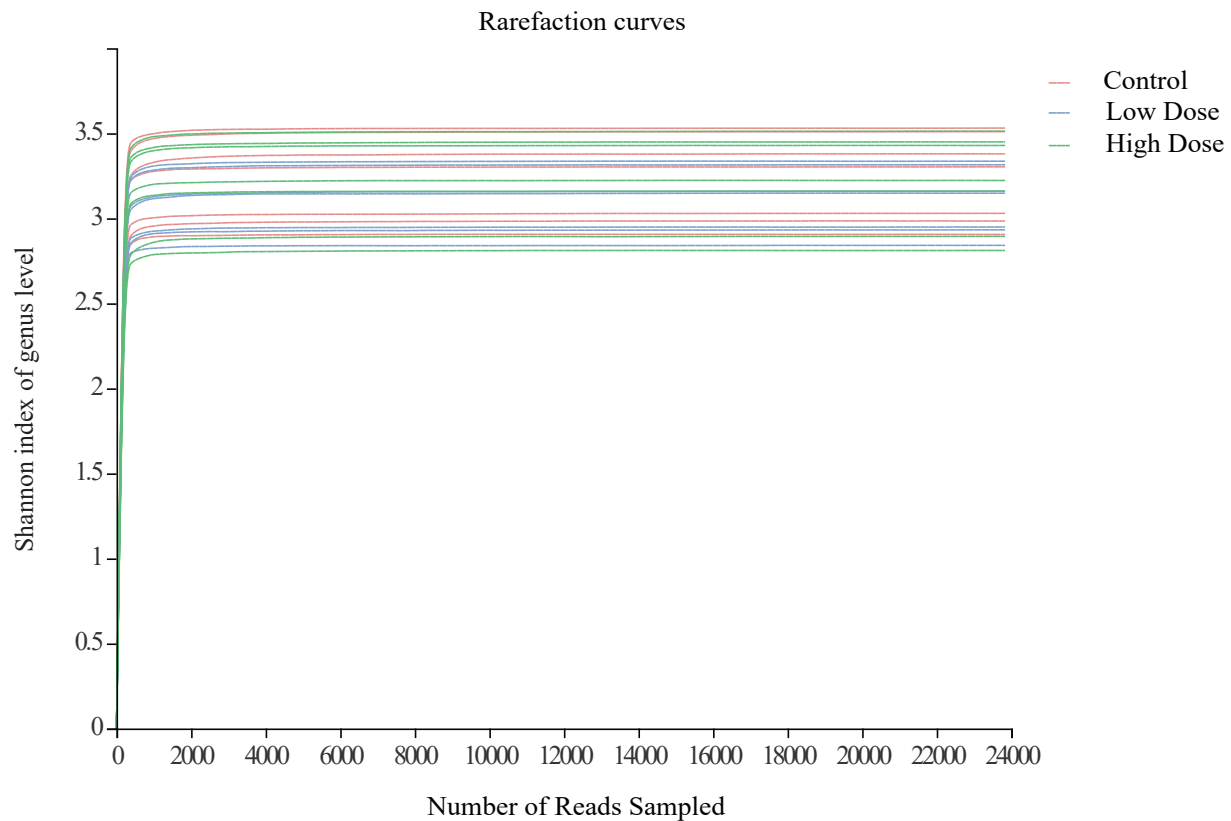

Supplement: Supplementary file 7 — Additional file 6: Figure S9. (a) Alpha diversity comparisons of the gut microbiomes including Ace, Chao, Sobs index, Shannon index, Shannoneven index, Simpson index, Simpsoneven index and Faith's phylogenetic diversity index which were analyzed using a Kruskal-Wallis H test and Tukey-Kramer post hoc test with 95% confidence level. Data are the mean ± s.d (n = 7). ns, no significant differences. (b) Rarefaction curves for Shannon indices at the genus level in the colon contents. Figure S10. (a) Bar charts of relative abundance at the family level in the control and treated groups. (b) Comparison of microbial genera between the high dose group and control. (c) Comparison of microbial families between high dose group and control. Significantly differentially abundant taxa were identified by the Wilcoxon rank-sum test. *p < 0.05; **p < 0.01. Figure S11. (a) Linear discriminant analysis score (log10) with a threshold value of 4 from phylum to genus level. (b) Differences in relative abundance of the variable genera in the colonic microbiota among three group. Each data represented the mean and SEM of relative abundance of each genus (n = 7). One-way analysis of variance and Duncan’s multiple comparison test to determine the statistical. Different letters in the same graph indicate significant statistical differences (p < 0.05). [file 40168_2022_1278_MOESM6_ESM.zip › Suppl. Fig. S9.pdf]
