# Supplementary material for: Antioxidant potential of Pediococcus pentosaceus strains from the sow milk bacterial collection in weaned piglets
Source: Microbiome. 2022 Jun 1;10:83. doi: 10.1186/s40168-022-01278-z (PMC9158380; doi:10.1186/s40168-022-01278-z)
Supplement: Supplementary file 5 — Additional file 4: Figure S5. The annotation and comparison of P. pentosaceus SMM914 genome. (a) The distribution of predicted CDSs of P. pentosaceus SMM914 in different categories of metabolic function by the online software RAST. (b) A full genome comparison analysis of P. pentosaceus SMM914 with other P. pentosaceus strains, including P. pentosaceus SRCM100194, P. pentosaceus GDIAS001, P. pentosaceus SL001 and P. pentosaceus SRCM102736, visualized by BRIG software. Colors display the percentage of sequence identity based on BLASTN. The two inner rings indicate the GC skew and the GC content. The innermost circle shows the genome coordinates. Supplementary Data 3. P. pentosaceus SMM914 genes and predicted proteins by Pfam protein database. Supplementary Table S1. Oxidative stress resistance genes found in P. pentosaceus SMM914. Supplementary Table S2. The annotation of antibiotic resistance genes in P. pentosaceus SMM914. Supplementary Table S3. The annotation of bacterial virulence factors in P. pentosaceus SMM914. [file 40168_2022_1278_MOESM4_ESM.zip › Supplementary table 1.docx]

Supplementary table 1. Oxidative stress resistance genes found in *P. pentosaceus* SMM914

| Gene id in SMM914 | Encoded proteins | Function |
| --- | --- | --- |
| GM001088  GM001820 | Exopolysaccharide | Possessing antioxidant activity by scavenging hydroxyl free radicals |
| GM001399  GM000924 | Thioredoxin | Maintaining the thiol/disulfide balance in cellular redox processes |
| GM000997 | Thioredoxin reductase | Regenerating reduced thioredoxins |
| GM000377 | NADH:flavin oxidoreductase / NADH oxidase family | Regulation of intracellular hydrogen peroxide concentration |
| GM001312 | NADH-dependent flavin reductase subunit 1 | Component of an enzyme that catalyzes the reduction of free flavins by NADH |
| GM000835  GM000280 | NADH oxidase | Catalyzing a four-electron reduction of molecular oxygen to water |
| GM001715 | Glutathione reductase | Maintaining redox homeostasis by catalyzing the conversion of glutathione disulfide to glutathione using NADPH as a cofactor |
| GM001123 | Lipoteichoic acid synthase 2 | Involved in the pathway lipoteichoic acid biosynthesis, which is part of cell wall biogenesis and could enhance the anti-oxidation activity |
