# Supplementary material for: Antioxidant potential of Pediococcus pentosaceus strains from the sow milk bacterial collection in weaned piglets
Source: Microbiome. 2022 Jun 1;10:83. doi: 10.1186/s40168-022-01278-z (PMC9158380; doi:10.1186/s40168-022-01278-z)
Supplement: Supplementary file 5 — Additional file 4: Figure S5. The annotation and comparison of P. pentosaceus SMM914 genome. (a) The distribution of predicted CDSs of P. pentosaceus SMM914 in different categories of metabolic function by the online software RAST. (b) A full genome comparison analysis of P. pentosaceus SMM914 with other P. pentosaceus strains, including P. pentosaceus SRCM100194, P. pentosaceus GDIAS001, P. pentosaceus SL001 and P. pentosaceus SRCM102736, visualized by BRIG software. Colors display the percentage of sequence identity based on BLASTN. The two inner rings indicate the GC skew and the GC content. The innermost circle shows the genome coordinates. Supplementary Data 3. P. pentosaceus SMM914 genes and predicted proteins by Pfam protein database. Supplementary Table S1. Oxidative stress resistance genes found in P. pentosaceus SMM914. Supplementary Table S2. The annotation of antibiotic resistance genes in P. pentosaceus SMM914. Supplementary Table S3. The annotation of bacterial virulence factors in P. pentosaceus SMM914. [file 40168_2022_1278_MOESM4_ESM.zip › Supplementary table 2.docx]

Supplementary table 2. The annotation of antibiotic resistance genes in *P. pentosaceus* SMM914

| Gene id in SMM914 | Identity | E value | Subject id | Resistance type | Antibiotic resistance | Original resistance type |
| --- | --- | --- | --- | --- | --- | --- |
| GM001786 | 61.30 | 8.8E-97 | ardb_320 | baca | bacitracin | baca |
| GM001958 | 99.80 | 0 | ardb_312 | tetm | tetracycline | tet |
| GM001973 | 99.10 | 2E-124 | ardb_1794 | erma | erythromycin | erm |
