# Supplementary material for: Antioxidant potential of Pediococcus pentosaceus strains from the sow milk bacterial collection in weaned piglets
Source: Microbiome. 2022 Jun 1;10:83. doi: 10.1186/s40168-022-01278-z (PMC9158380; doi:10.1186/s40168-022-01278-z)
Supplement: Supplementary file 5 — Additional file 4: Figure S5. The annotation and comparison of P. pentosaceus SMM914 genome. (a) The distribution of predicted CDSs of P. pentosaceus SMM914 in different categories of metabolic function by the online software RAST. (b) A full genome comparison analysis of P. pentosaceus SMM914 with other P. pentosaceus strains, including P. pentosaceus SRCM100194, P. pentosaceus GDIAS001, P. pentosaceus SL001 and P. pentosaceus SRCM102736, visualized by BRIG software. Colors display the percentage of sequence identity based on BLASTN. The two inner rings indicate the GC skew and the GC content. The innermost circle shows the genome coordinates. Supplementary Data 3. P. pentosaceus SMM914 genes and predicted proteins by Pfam protein database. Supplementary Table S1. Oxidative stress resistance genes found in P. pentosaceus SMM914. Supplementary Table S2. The annotation of antibiotic resistance genes in P. pentosaceus SMM914. Supplementary Table S3. The annotation of bacterial virulence factors in P. pentosaceus SMM914. [file 40168_2022_1278_MOESM4_ESM.zip › Supplementary table 3.docx]

Supplementary table 3. The annotation of bacterial virulence factors in *P. pentosaceus* SMM914

| VF name | Gene id in SMM914 | Identity | E value | VFDB id | Related genes |
| --- | --- | --- | --- | --- | --- |
| Capsule | GM000023 | 41.30 | 1.7E-13 | VFG037973 | A1S_0057 capsular polysaccharide synthesis enzyme |
| Capsule | GM000281 | 42.90 | 4.3E-17 | VFG037973 | A1S_0057 capsular polysaccharide synthesis enzyme |
| Capsule | GM000317 | 41.10 | 2.9E-62 | VFG002180 | cpsK ABC transporter, permease protein |
| Capsule | GM000318 | 49.90 | 0 | VFG002181 | cpsJ ABC transporter, ATP-binding protein |
| Capsule | GM000321 | 46.80 | 2.1E-90 | VFG002188 | cpsC teichoic acid biosynthesis protein, putative |
| Capsule | GM000558 | 50.80 | 4.2E-69 | VFG002189 | cpsB phosphatidate cytidylyltransferase |
| Capsule | GM000559 | 64.00 | 9.1E-87 | VFG045688 | uppS undecaprenyl diphosphate synthase |
| Capsule | GM000661 | 42.40 | 3.5E-42 | VFG046604 | Fphi_1467 ribulose-phosphate 3-epimerase |
| Capsule | GM000950 | 43.90 | 5E-30 | VFG016532 | oppF oligopeptide ABC transporter, permease component |
| Capsule | GM000951 | 47.30 | 0 | VFG016532 | oppF oligopeptide ABC transporter, permease component |
| Capsule | GM000976 | 47.90 | 1.7E-95 | VFG018674 | SGO_1723 RgpG |
| Capsule | GM000996 | 71.30 | 5.8E-117 | VFG005874 | hasC UTP--glucose-1-phosphate uridylyltransferase |
| Capsule | GM001091 | 60.10 | 0 | VFG005923 | SSU05_0569 Cps2F |
| Capsule | GM001097 | 72.70 | 5E-121 | VFG006052 | rmlA glucose-1-phosphate thymidyl transferase |
| Capsule | GM001099 | 81.90 | 0 | VFG006022 | STER_1222 dTDP-D-glucose 4,6-dehydratase |
| Capsule | GM001100 | 58.40 | 1.2E-87 | VFG006089 | STER_1444 dTDP-4-dehydrorhamnose reductase |
| Capsule | GM001101 | 62.40 | 8.9E-176 | VFG005844 | STER_1057 Polysaccharide Transporter, PST family |
| Capsule | GM001116 | 58.90 | 1E-121 | VFG001373 | cps4I UDP-N-acetylglucosamine-2-epimerase |
| Capsule | GM001536 | 41.10 | 5.6E-86 | VFG002188 | cpsC teichoic acid biosynthesis protein, putative |
| Polysaccharide capsule | GM001087 | 40.30 | 1.6E-41 | VFG016385 | BC5278 Chain length regulator capsular polysaccharide biosynthesis |
| Polysaccharide capsule | GM001088 | 46.60 | 2.2E-53 | VFG016404 | BCE_5398 capsular exopolysaccharide family protein |
| Polysaccharide capsule | GM001090 | 55.80 | 1.4E-67 | VFG016432 | epsE sugar transferase |
| Polysaccharide capsule | GM001110 | 42.30 | 3E-56 | VFG016423 | lytR transcription antiterminator |
| Polysaccharide capsule | GM001189 | 58.10 | 7.8E-110 | VFG016308 | galE UDP-glucose 4-epimerase |
| Polysaccharide capsule | GM001322 | 51.30 | 1.3E-90 | VFG016424 | manA mannose-6-phosphate isomerase |
| Polysaccharide capsule | GM001750 | 55.70 | 1.8E-106 | VFG016308 | galE UDP-glucose 4-epimerase |
| Hcp secretion island-1 encoded type VI secretion system (H-T6SS) | GM000054 | 41.00 | 0 | VFG015379 | PSPA7_0144 probable ATP-binding component of ABC transporter |
| Hcp secretion island-1 encoded type VI secretion system (H-T6SS) | GM000178 | 40.20 | 1.7E-27 | VFG015382 | Psyr_2628 ABC transporter |
| Hcp secretion island-1 encoded type VI secretion system (H-T6SS) | GM001552 | 40.30 | 3.4E-141 | VFG015498 | clpV1 ATPase AAA-2 domain protein |
| LPS | GM000640 | 42.00 | 5.6E-25 | VFG011402 | fabZ 3R-hydroxymyristoyl ACP dehydratase |
| LPS | GM000654 | 40.30 | 2.6E-11 | VFG011430 | acpXL acyl carrier protein |
| LPS | GM000843 | 42.70 | 6.5E-94 | VFG047258 | FN3523_0439 N-acetylglucosamine-1-phosphate uridyltransferase/glucosamine-1-phosphate N-acetyltransferase |
| RegX3 | GM000122 | 49.80 | 9.8E-59 | VFG031738 | regX3 Sensory transduction protein RegX3 |
| RegX3 | GM000983 | 40.20 | 4.1E-49 | VFG031729 | regX3 two component transcriptional regulator |
| RegX3 | GM001305 | 42.00 | 4.6E-40 | VFG031738 | regX3 Sensory transduction protein RegX3 |
| Trehalose-recycling ABC transporter | GM000266 | 41.00 | 7.8E-48 | VFG030692 | sugC sugar ABC transporter |
| Trehalose-recycling ABC transporter | GM001371 | 42.70 | 7.6E-34 | VFG030675 | sugC SugC |
| Trehalose-recycling ABC transporter | GM001734 | 47.00 | 2.1E-57 | VFG030684 | sugC ABC transporter--like protein |
| ClpE | GM000458 | 46.30 | 5.8E-163 | VFG000080 | clpE ATP-dependent protease |
| ClpE | GM001126 | 60.10 | 1.1E-244 | VFG000080 | clpE ATP-dependent protease |
| Fibronectin-binding protein | GM000496 | 45.50 | 3.2E-135 | VFG031927 | fbpA hypothetical protein |
| Fibronectin-binding protein | GM001563 | 40.70 | 2.7E-33 | VFG043456 | scpB segregation and condensation protein B |
| Nucleoside diphosphate kinase | GM000277 | 46.70 | 0 | VFG031465 | ndk nucleoside diphosphate kinase |
| Nucleoside diphosphate kinase | GM001596 | 46.00 | 1.6E-27 | VFG031486 | ndk nucleoside diphosphate kinase |
| Pyrimidine biosynthesis | GM000865 | 41.80 | 1.4E-75 | VFG047719 | carA carbamoyl phosphate synthase small subunit |
| Pyrimidine biosynthesis | GM000866 | 46.80 | 5.5E-266 | VFG047710 | FN3523_0021 carbamoyl-phosphate synthase large chain |
| T3SS | GM001893 | 41.10 | 0 | VFG042130 | mlr6326 putative DNA invertase |
| T3SS | GM001907 | 41.10 | 3.1E-22 | VFG042130 | mlr6326 putative DNA invertase |
| T4SS effectors | GM000554 | 40.60 | 0 | VFG039487 | CbuG_0575 hypothetical protein |
| T4SS effectors | GM001417 | 46.50 | 1.5E-57 | VFG039536 | CBU_1566 Coxiella Dot/Icm type IVB secretion system translocated effector |
| Hemolysin | GM000674 | 55.30 | 1.5E-79 | VFG012174 | Cthe_0827 hemolysin A |
| Hemolysin III | GM000035 | 41.90 | 7.3E-45 | VFG016229 | hlyIII Hemolysin III |
| (p)ppGpp synthesis and hydrolysis | GM001528 | 40.30 | 1.7E-168 | VFG022965 | relA GTP pyrophosphokinase |
| Accessory secretion factor | GM000981 | 40.20 | 8.3E-118 | VFG009228 | secA2 ATPase SecA2 |
| AI-2 | GM000283 | 42.10 | 1.9E-29 | VFG018241 | luxS S-ribosylhomocysteinase |
| Capsular polysaccharide | GM001098 | 47.50 | 5.3E-42 | VFG007663 | rmlC dTDP-6-deoxy-D-xylo-4-hexulose-3,5-epimerase |
| ClpC | GM001231 | 55.70 | 0 | VFG000079 | clpC endopeptidase Clp ATP-binding chain C |
| ClpP | GM001009 | 71.10 | 0 | VFG000077 | clpP ATP-dependent Clp protease proteolytic subunit |
| D-alanine-polyphosphoribitol ligase | GM000329 | 50.40 | 9.7E-136 | VFG032791 | dltA D-alanine--polyphosphoribitol ligase subunit 1 |
| Dot/Icm | GM001540 | 42.20 | 1.8E-29 | VFG045566 | lpg2359 Dot/Icm type IV secretion system effector |
| EF-Tu | GM001507 | 71.30 | 2.2E-167 | VFG016490 | tuf translation elongation factor Tu |
| Exopolysaccharide | GM001030 | 43.00 | 3.1E-93 | VFG013515 | mrsA/glmM predicted phosphomannomutase |
| FAS-II | GM000635 | 40.30 | 0 | VFG009141 | kasB 3-oxoacyl-acyl-carrier-protein synthase |
| Glutamine synthesis | GM000692 | 42.70 | 0 | VFG026433 | glnA1 glutamine synthetase |
| GroEL | GM000974 | 67.40 | 5.5E-206 | VFG012103 | groEL chaperonin GroEL |
| GtcA | GM000313 | 40.90 | 1E-19 | VFG032845 | gtcA wall teichoic acid glycosylation protein GtcA |
| histone-like protein (Hlp)/laminin-binding protein (LBP) | GM001571 | 45.50 | 0 | VFG043551 | ML1683 histone-like protein |
| Lipoprotein diacylglyceryl transferase | GM000994 | 61.00 | 8.1E-92 | VFG032466 | lgt putative prolipoprotein diacylglyceryl transferase |
| Lipoprotein-specific signal peptidase II | GM000501 | 40.80 | 5.4E-26 | VFG006811 | lspA signal peptidase II |
| LisR/LisK | GM000716 | 71.10 | 4.3E-91 | VFG006826 | lisR two-component response regulator |
| LOS | GM001401 | 46.80 | 1.2E-36 | VFG013269 | orfM putative deoxyribonucleotide triphosphate pyrophosphatase |
| LPS glucosylation | GM000314 | 41.20 | 1.7E-63 | VFG012939 | gtrB bactoprenol glucosyl transferase |
| Lysine synthesis | GM001686 | 42.40 | 6.8E-90 | VFG009388 | lysA diaminopimelate decarboxylase |
| Magnesium transport | GM001701 | 44.60 | 9E-25 | VFG022673 | mgtC Mg2+ transport P-type ATPase C MgtC |
| MOMP | GM000544 | 59.70 | 5E-203 | VFG043573 | CT396 molecular chaperone DnaK |
| Oligopeptide-binding protein | GM000947 | 41.70 | 6.3E-125 | VFG032255 | oppA oligopeptide ABC transporter substrate-binding protein |
| PDH-B | GM000147 | 45.10 | 4.5E-78 | VFG016506 | pdhB pyruvate dehydrogenase E1 component, beta subunit |
| PhoP/R | GM000205 | 40.40 | 1.5E-35 | VFG022904 | phoP two-component system response phosphate regulon transcriptional regulator, PhoP |
| Phytotoxin phaseolotoxin | GM000290 | 43.90 | 2.9E-72 | VFG015903 | argK phaseolotoxin-insensitive ornithine carbamoyltransferase |
| Polar flagella | GM000636 | 44.20 | 1.9E-49 | VFG038840 | flmH 3-oxoacyl-ACP reductase |
| Serine protease | GM000127 | 50.80 | 6.2E-96 | VFG005533 | htrA/degP serine protease HtrA |
| Serine-threonine phosphatase | GM000664 | 42.60 | 4.8E-51 | VFG006797 | stp serine/threonine protein phosphatase family protein |
| Sigma A | GM001549 | 55.00 | 1.3E-97 | VFG009718 | sigA/rpoV sigma factor MysA |
| Streptococcal enolase | GM001015 | 71.50 | 1.3E-176 | VFG005582 | eno Enolase, putative |
| Streptococcal lipoprotein rotamase A | GM001754 | 47.80 | 8.5E-48 | VFG019080 | slrA peptidyl-prolyl cis-trans isomerase, cyclophilin-type |
| Streptococcal plasmin receptor/GAPDH | GM001012 | 58.80 | 1.4E-109 | VFG005356 | plr/gapA glyceraldehyde-3-phosphate dehydrogenase, type I |
| Trigger factor | GM001508 | 57.40 | 0 | VFG005535 | tig/ropA trigger factor |
